# Lipopolysaccharide-Induced Differential Expression of miRNAs in Male and Female *Rhipicephalus haemaphysaloides* Ticks

**DOI:** 10.1371/journal.pone.0139241

**Published:** 2015-10-02

**Authors:** Fangfang Wang, Haiyan Gong, Houshuang Zhang, Yongzhi Zhou, Jie Cao, Jinlin Zhou

**Affiliations:** 1 Key Laboratory of Animal Parasitology of Ministry of Agriculture, Shanghai Veterinary Research Institute, Chinese Academy of Agricultural Sciences, Shanghai, China; 2 Jiangsu Co-innovation Center for Prevention and Control of Important Animal Infectious Diseases and Zoonoses, Yangzhou, China; University of Minnesota, UNITED STATES

## Abstract

Lipopolysaccharide (LPS) stimulates the innate immune response in arthropods. In tick vectors, LPS activates expression of immune genes, including those for antibacterial peptides. miRNAs are 21–24 nt non-coding small RNAs that regulate target mRNAs at the post-transcriptional level. However, our understanding of tick innate immunity is limited to a few cellular immune reactions and some characterized immune molecules. Moreover, there is little information on the regulation of the immune system in ticks by miRNA. Therefore, this study aimed to analyze the differential expression of miRNAs in male and female ticks after LPS injection. LPS was injected into male and female *Rhipicephalus haemaphysaloides* ticks to stimulate immune response, with phosphate buffered saline (PBS)-injected ticks as negative controls. miRNAs from each group were sequenced and analyzed. In the PBS- and LPS-injected female ticks, 11.46 and 12.82 million reads of 18–30 nt were obtained respectively. There were 13.92 and 15.29 million reads of 18–30 nt obtained in the PBS- and LPS-injected male ticks, respectively. Expression of miRNAs in male ticks was greater than that in female ticks. There were 955 and 984 conserved miRNA families in the PBS- and LPS-injected female ticks, respectively, and correspondingly 1684 and 1552 conserved miRNA families in male ticks. Nine novel miRNAs were detected as common miRNAs in two or more tested samples. There were 37 known miRNAs up-regulated >10-fold and 33 down-regulated >10-fold in LPS-injected female ticks; and correspondingly 52 and 59 miRNAs in male ticks. Differential expression of miRNAs in PBS- and LPS-injected samples supports their involvement in the regulation of innate immunity. These data provide an important resource for more detailed functional analysis of miRNAs in this species.

## Introduction

Ticks are obligate hematophagous ectoparasites with a global distribution, and have veterinary and medical importance. They belong to the Phylum Arthropoda, Class Arachnida, Subclass Acari, Order Parasitoformes and Suborder Ixodida [1]. They transmit a variety of disease agents including viruses, bacteria and protozoa to humans, domesticated animals and wildlife [2]. Why do ticks themselves escape from attack and damage by those pathogens? This may be associated with the innate immune system of ticks. Ticks lack an adaptive immune system and their defense against potential pathogens relies on a network of cellular immune reactions and various humoral factors to recognize and eliminate pathogens [3]. Hemocoel, midgut, salivary glands and the fat body in ticks are major organs responsible for the innate immunity by secreting antimicrobial peptides (AMPs) including defensins. Several members of the defensin family have been identified from *Ixodes scapularis* [4]. Lipopolysaccharide (LPS) elicits immune-related gene expression in the fat body of arthropods such as *Bombyx mori* [5], consistent with it inducing expression of defensin in *Haemaphysalis longicornis* [6]. However, how these immune-related molecules are regulated in ticks remains unknown. Recently, 73 miRNAs in *Drosophila melanogaster* were studied and thought to be immune-miRNAs, with their potential target genes components of the Toll, Tmd, melanization, C-Jun N-terminal kinase (JNK) and Janus kinase (JAK)/ signal transducer and activator of transcription (STAT) pathways; six of which probably participate in the immune response [7]. In addition, conserved miRNA miR-8 in fat body regulates AMP expression in innate immune homeostasis in *Drosophila*, while the levels of AMPs such as drosomycin and diptericin are significantly increased in miR-8 null animals in non-pathogen-stimulated conditions [8]. The main components of the Toll immune pathway, Toll and Dorsal, are targets of miR-8 [9]. Meanwhile, Etebari and Asgari described miR-8 targeted Serpin 27, which regulates activation of the Toll pathway and prophenoloxidase, which is involved in the melanization response in the insect *Plutella xylostella* [10].

miRNAs are non-coding small RNA (sRNA) molecules of 21–24 nt that negatively regulate gene expression at the post-transcriptional level, via base pairing to target sites with mRNA [11]. miRNA genes are independent transcriptional units, known as the primary miRNA (pri-miRNA) with one or more stem-loops. The pri-miRNA is cleaved in the nucleus by RNA polymerase II enzyme into ~70-nt precursor miRNA (pre-miRNA) that consists of an imperfect stem-loop structure. The pre-miRNA is then transported into the cytoplasm by Exportin 5 and Ran-GTP, which is diced by RNase III enzyme to form the miRNA-miRNA* duplex. Finally, mature miRNA is incorporated into the RNA-induced silencing complex, resulting in translational repression and mRNA degradation [12]. As the gene regulatory molecules, miRNAs participate in a diverse range of biological processes such as development, proliferation, immunity and stress responses [13]. Since 2008, 37 miRNAs in *I*. *scapularis* have been reported [14]. 36 novel miRNAs were then identified in *Rhipicephalus microplus*; 12 of which were conserved in *I*. *scapularis* [15]. These miRNAs show a variety of expression profiles, with the evolutionarily conserved miRNAs ubiquitously expressed in all life stages at various levels, while the novel tick-specific miRNAs are mostly limited to particular life stages and/or tick organs [15]. In addition, distinct miRNA profiles in the salivary glands of *H*. *longicornis* indicate that miRNAs are related to tick blood feeding [16], while there has been no study on specific miRNA profiles related to tick innate immunity.

The tick species *R*. *haemaphysaloides* is widespread in China and other South East Asian countries and is a vector of animal babesiosis and human Kyasanur Forest disease [4]. To provide new insights into innate immunity of ticks and to expand our knowledge of miRNAs, sRNA transcriptome profiles derived from LPS- and phosphate buffered saline (PBS)-injected female and male *R*. *haemaphysaloides* ticks were studied and the differences in expression of miRNAs were analyzed.

## Materials and Methods

### Ethics Statement

The protocols were approved by the Institutional Animal Care and Use Committee of the Shanghai Veterinary Research Institute, and authorized by the Animal Ethical Committee of Shanghai Veterinary Research Institute.

### Tick sampling and total RNA preparation

*R*. *haemaphysaloides* were maintained in our laboratory at the Chinese Academy of Agricultural Sciences (Shanghai, China) [17]. In detail, a cloth bag was fixed on the ear of a rabbit by adhesive bandage, and ticks were kept in the closed bag until they were engorged. These ticks were then kept in an incubator at 25°C with a humidity of 95% for oviposition or moult. LPS (Sigma, St Louis, MO, USA) was diluted to 0.2 mg/ml and stored at –20°C until use. One hundred unfed adult female ticks were injected with 0.1 μg/ml LPS (0.5 μl each), or the same volume of PBS (50 ticks for each group) using a microsyringe through the joint between the coxa IV and the sternal plate. After being kept for 24 h in a dark incubator, 50 injected female ticks from each group were ground to powder in liquid nitrogen using a sterile mortar and pestle. One hundred adult males were also treated as described above. Total RNA was extracted using TRIzol reagent (Invitrogen, Carlsbad, CA, USA). The quality of the total RNA was examined by standard agarose gel electrophoresis, and the concentration was determined using a BioPhotometer (GE, Fairfield, CT, USA). The extracted total RNA samples were stored at –80°C until deep sequencing or quantitative (q)RT-PCR.

### sRNA isolation and high-throughput sequencing

RNA fragments of 18–30 bases were isolated and purified by Novex 15% TBE-Urea gel, and then 5ʹ and 3ʹ adaptors (Illumina, San Diego, CA,USA) were added to the ends of fragments. RT-PCR was performed using an Invitrogen kit. The fragments were purified using a 6% TBE PAGE and used for high-through-put sequencing using a Solexa sequencer at BGI Genomics Institute (Beijing, China).

### Sequence analysis and identification of homologs of conserved miRNA

After removing the adaptor sequences and redundant reads of <18 nt, the clean reads were first screened against GenBank and the Rfam databases (version 9.0; http://www.sanger.ac.uk/software/Rfam/mirna) to remove non-coding RNAs such as rRNAs, tRNAs, snRNAs, snoRNAs and other ncRNAs. Before the remaining reads were searched against the Sanger miRBase (version 17.0) to identify conserved miRNAs, the repetitive sequences were eliminated using Repeat Masker (http://www.repeatmasker.org), and exons and introns of mRNA were masked. Based on miRNA nomenclature, reads with high homology to known miRNAs from other organisms were classified into the same family (≤2 mismatches) [18]. Reads matching none of these databases were marked as unannotated. Clean reads were then mapped onto the *I*. *scapularis* genome (http://iscapularis.vectorbase.org/) to analyze their expression and distribution on the genome using the Short Oligo-nucleotide Analysis Package (SOAP) [19].

### Differential expression analysis of miRNAs

The differential expression of known miRNAs in the four groups was compared to identify some differentially expressed miRNAs and plotted as the log_2_ ratio and a scatter plot. The procedures were as follows:

1. Normalize the expression of miRNAs in the control and treatment samples to obtain expression of transcripts per million. Normalization formula: normalized miRNA expression = (actual miRNA count/total count of clean reads) × 1,000,000
2. Calculate the fold-change and P-value from the normalized expression. Generate the log_2_ ratio plot and scatter plot. Fold-change formula: fold-change = log_2_ (treatment/control)

P-value formula:

$$p\left(x/y\right)={\left(\frac{N_{2}}{N}\right)}^{y}\frac{\left(x+y\right)!}{x!y!{\left(1+\frac{N_{2}}{N}\right)}^{\left(x+y+1\right)}}\begin{array}{l} C\left(y\leq y_{\text{min}}/x\right)=\sum \begin{array}{l} y\leq y_{\text{min}}\\ y-0 \end{array}p\left(y/x\right)\\ D\left(y\geq y_{\text{max}}/x\right)=\sum \begin{array}{l} \infty \\ y\geq y_{\text{max}} \end{array}p\left(y/x\right) \end{array}$$

### Verification of the miRNAs by qRT-PCR

Five miRNAs with down-regulation in LPS-injected female ticks were randomly selected for qRT-PCR. cDNAs were synthesized from the total RNAs of LPS- and PBS-injected female ticks using miScript II RT kit (Qiagen, Hilden, Germany). PCR was performed on an Applied Biosystems 7500 using miScript SYBR Green PCR Kit (Qiagen) [20], using the following PCR primers. miR-184, 5ʹ-TGGACGGAGAACTGATAAGGGC-3ʹ; miR-79-3p, 5-GCGCCATAAAGCTAGGTTACCAAAG ʹ -3ʹ; miR-71-5p, 5ʹ-GCGTGAAAGACATGGGTAGTGAG-3ʹ; miR-133-3p, 5ʹ-GCGCCTTGGTCCCCTTCAA-3ʹ; and miR-1-3p, 5ʹ-GCGCCTGGAATGTAAAGAAGTATGT-3ʹ. Tick elongation factor 1α (ELF1A) was used as the internal control on account of its stability in *Rhipicephalus*. *microplus* and *Rhipicephalus*. *appendiculatus* ticks, as well as *R*. *haemaphysaloides* [21] (forward primer: 5ʹ-CGTCTACAAGATTGGTGGCATT-3ʹ; and reverse primer: 5ʹ-CTCAGTGGTCAGGTTGGCAG-3ʹ). All reactions were performed in triplicate. The expression of miRNAs was normalized to that of ELF1A, and the relative abundance of miRNAs was calculated using 2^–ΔCt^ method.

## Results

### Profile characteristics of sRNA pools in libraries

**Fig 1:**
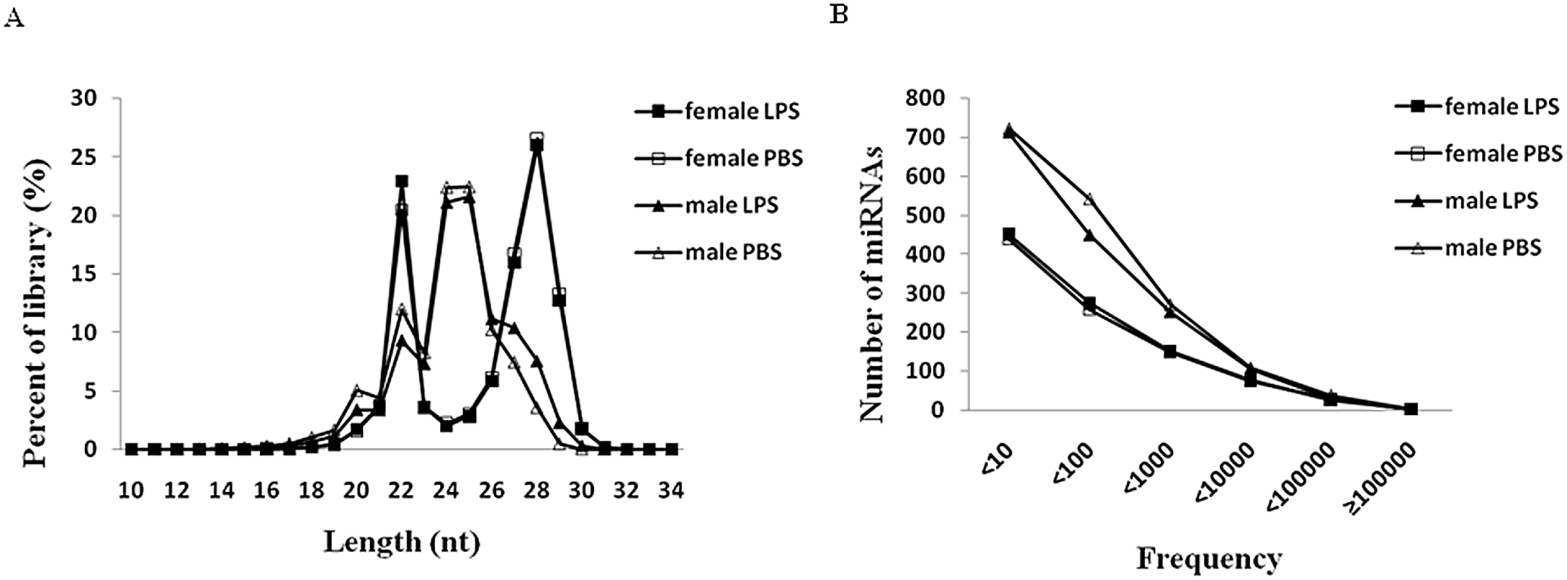
Deep sequencing of sRNA profiles in the four samples. A) Size distributions of sRNA libraries from the four samples. B) Frequencies of read counts for known miRNAs present in the LPS- and PBS-injected female and male samples.

To identify miRNAs from *R*. *haemaphysaloides* and to obtain the differentially expressed miRNAs related to innate immunity in the four samples, raw data from the four sRNA pools were obtained using the latest Solexa sequencing technology. Data of the miRNAs were deposited in the DNA databank of Japan with accession number: PRJDB3508. This approach generated >54 million short reads derived from the tick sRNA transcriptome in the LPS- and PBS-injected female and male ticks (Table 1). Before collecting these sRNAs and analyzing their size distribution, the low quality reads were filtered and the 3ʹ- and 5ʹ-adaptor sequences were cleaned up. Deep sequencing yielded about 11.53, 12.89, 14.11 and 15.41 million reads with high quality from PBS- and LPS-treated female and PBS- and LPS-treated male ticks, respectively. Based on the size distribution of the total reads, LPS- and PBS-treated female miRNA profiles showed a different bimodal distribution, with one peak around 20–22 nt representing miRNAs and another peak around 26–28 nt representing longer piRNA-like sRNAs. The majority of PBS- and LPS-injected sRNAs from male ticks were in the range of 22–26 nt, with 22 and 24 nt as the two major size groups (Fig 1A). The peak at 20–22 nt was higher in the miRNA profile for LPS- than PBS-treated female ticks, suggesting that sRNAs in the LPS-treated female ticks were more abundant and diverse. However, the peak at 22–26 nt did not significantly differ between LPS- and PBS-treated male ticks.

**Table 1:** Breakdown of the total number of reads obtained for each library, and short read statistics for evolutionarily conserved and novel *R*. *haemaphysaloides* miRNAs. The clean reads are obtained after removal of contaminant reads.

Sample	Total reads	Clean reads	Evolutionary conserved miRNAs	Novel miRNAs	Total miRNAs
**Male LPS**	15,492,326	15,286,372	3,092,772	8,242	3,101,014
**Male PBS**	14,189,315	13,918,024	3,929,669	104	3,929,773
**Female LPS**	12,943,158	12,820,011	6,265,221	1,898	6,267,119
**Female PBS**	11,581,574	11,459,406	5,070,412	562	5,070,974
**Total**	54,206,373	53,483,813	18,358,074	10,806	18,368,880

In the next step, reads of <18 nt were removed, and a total of 11.46 million clean reads remained with 2.10 million (18.32%) unique sequences in the PBS-injected female profile. Similarly, a total of 12.82 million clean reads remained with 2.18 million (17.00%) unique sequences in the LPS-injected female group. A total of 15.29 million clean reads remained with 2.47 million (16.15%) unique sRNAs in the LPS-treated male group, while a total of 13.92 million clean reads remained with 2.05 million (14.73%) unique sRNAs in the PBS-injected male group. The number of male and female sRNAs increased after LPS induction in terms of both unique and total sRNAs (Tables 2 and 3). Among the clean reads of PBS- and LPS-injected samples, only 18.11 and 20.26% were perfectly mapped to the *I*. *scapularis* genome, including 0.96 and 0.89% unique sequences, respectively. Only 10.44 and 7.27% were perfectly mapped to the *I*. *scapularis* genome, including 1.14 and 0.97% unique sequences from the clean reads of PBS- and LPS-treated male samples, respectively.

**Table 2:** Common and specific sRNAs from LPS- and PBS-injected female samples. The number of unique or total common sRNAs between LPS- and PBS-injected female ticks and the percentage are reported. The number of unique or total specific sRNA and the percentage are also reported.

Class	Unique sRNAs	%	Total sRNAs	%
**Total_sRNAs**	3644020	100	24279417	100
**Common**	642670	17.64	20921599	86.17
**Female LPS specific**	1539891	42.26	1729543	7.12
**Female PBS specific**	1461459	40.11	1628275	6.71

**Table 3:** Common and specific sRNAs from LPS- and PBS-injected male samples. The number of unique or total common sRNAs between LPS- and PBS-injected male ticks and the percentage are reported. The number of unique or total specific sRNA and the percentage are also reported.

Class	Unique sRNAs	%	Total sRNAs	%
**Total_sRNAs**	3923329	100	29204396	100
**Common**	589362	15.02	25312131	86.67
**Male LPS specific**	1877122	47.85	2283343	7.82
**Male PBS specific**	1456845	37.13	1608922	5.51

Among the clean reads, 1.29 and 1.16% were ncRNAs in the PBS- and LPS-treated female samples, respectively, including rRNAs, tRNAs, snRNAs and snoRNAs. We aligned sequencing reads to known miRNAs and miRNA* strands (also termed guide and passenger strand, respectively) presented in miRBase version 17.0 (http://www.mirbase.org/). The proportion of known miRNAs in PBS-injected female ticks was 25.58% from 2,930,960 reads, which included 23,261 unique sequences. These reads from PBS-injected female samples corresponded to 60 distinct pre-miRNAs. The sRNAs matching known miRNAs from PBS-injected female ticks are shown in S1 Table. Similarly, the proportion of known miRNAs in LPS-injected tick samples was 27.84% in 3,569,124 reads, which included 21,492 unique sequences. The reads from LPS-injected female samples corresponded to 58 distinct pre-miRNAs. The sRNAs matching known miRNAs from LPS-injected female ticks are shown in S2 Table. Apart from the miRNA, rRNA and the repeats mentioned above, 73.11 and 70.97% of PBS- and LPS-injected female tick sequences had no matches and were marked as unannotated (Table 4).

**Table 4:** Summary of clean reads that match various RNAs from LPS- and PBS-injected female samples. Number of unique or total sRNA reads in each sample matched to all categories of RNA. ‘Unann’ indicates unannotated reads and ‘repeat’ indicates repeat-associated sRNA.

Category	Unique sRNAs		Total sRNAs	
	Female LPS (%)	Female PBS (%)	Female LPS (%)	Female PBS (%)
**Total**	2182561(100)	2104129(100)	12820011(100)	11459406(100)
**Exon_antisense**	56(0.00)	33(0.00)	128(0.00)	91(0.00)
**Exon_sense**	174(0.01)	169(0.01)	241(0.00)	234(0.00)
**Intron_antisense**	314(0.01)	322(0.02)	790(0.01)	641(0.01)
**Intron_sense**	647(0.03)	559(0.03)	2064(0.02)	1741(0.02)
**miRNA**	21492(0.98)	23261(1.11)	3569124(27.84)	2930960(25.58)
**rRNA**	21766(1.00)	19891(0.95)	95259(0.74)	101072(0.88)
**repeat**	84(0.00)	97(0.00)	218(0.00)	258(0.00)
**snRNA**	655(0.03)	652(0.03)	1292(0.01)	1272(0.01)
**snoRNA**	44(0.00)	48(0.00)	64(0.00)	69(0.00)
**tRNA**	7772(0.36)	7235(0.34)	52073(0.41)	45331(0.40)
**unann**	2129557(97.57)	2051862(97.52)	9098758(70.97)	8377737(73.11)

Among the clean reads, 0.84 and 0.93% were ncRNAs in the PBS- and LPS-injected male samples, respectively. The proportion of known miRNAs in PBS-injected male samples was 17.57% from 2,445,891 reads, which included 45,511 unique sequences. These reads from PBS-injected male samples corresponded to 52 distinct pre-miRNAs. The sRNAs matching known miRNAs from PBS-injected male ticks are shown in S3 Table. Similarly, the proportion of known miRNAs in LPS-injected male tick samples was 13.04% from 1,993,554 reads, which included 42,511 unique sequences. These reads from LPS-injected male samples corresponded to 53 distinct pre-miRNAs. The sRNAs matching known miRNAs from LPS-injected male ticks are shown in S4 Table. Apart from the miRNA, rRNA and repeats mentioned above, 81.57% (PBS-injected male ticks) and 85.98% (LPS-injected male ticks) sequences had no matches and were marked as unannotated (Table 5).

**Table 5:** Summary of clean reads that match various RNAs from LPS- and PBS-injected male samples. Number of unique or total sRNA reads in each sample matched to all categories of RNA. ‘Unann’ indicates unannotated reads and ‘repeat’ indicates repeat-associated sRNA.

Category	Unique sRNAs		Total sRNAs	
	male LPS (%)	male PBS (%)	male LPS (%)	male PBS (%)
**Total**	2466484(100)	2046207(100)	15286372(100)	13918024(100)
**Exon_antisense**	89(0.00)	82(0.00)	124(0.00)	114(0.00)
**Exon_sense**	208(0.01)	189(0.01)	261(0.00)	261(0.00)
**Intron_antisense**	626(0.03)	672(0.03)	4989(0.03)	997(0.01)
**Intron_sense**	1064(0.04)	1082(0.05)	2335(0.02)	2137(0.02)
**miRNA**	42511(1.72)	45511(2.22)	1993554(13.04)	2445891(17.57)
**rRNA**	20742(0.84)	21608(1.06)	97623(0.64)	77781(0.56)
**repeat**	210(0.01)	162(0.01)	515(0.00)	230(0.00)
**snRNA**	822(0.03)	773(0.04)	2389(0.02)	2202(0.02)
**snoRNA**	102(0.00)	110(0.01)	121(0.00)	125(0.00)
**tRNA**	6274(0.25)	5435(0.27)	40907(0.27)	35798(0.26)
**unann**	2393836(97.05)	1970583(96.30)	13143554(85.98)	11352488(81.57)

### Identification of conserved miRNAs in the four tick samples

Based on the number of reads, the miRNA expression levels varied greatly and spanned five orders of magnitude for the four tick samples (Fig 1B). The majority of miRNAs (>96%) were sequenced 1–10,000 times (Fig 1B). Overall, there were 955 conserved miRNAs in the PBS-injected female ticks, wherein 698 miRNAs had copy numbers <100, of which 153 had only one copy. We found that 150 miRNAs had copy numbers of 100–1,000, and 75 had copy numbers of 1,000–10,000. Furthermore, only 32 miRNAs had copy numbers of >10,000. In the LPS-injected female samples, there were 984 conserved miRNAs; among these, 725 miRNAs had copy numbers of <100, of which 156 had only one copy. There were 152 miRNAs with copy numbers of 100–1,000, and only 77 had copy numbers of 1,000–10,000. Moreover, only 30 miRNAs had copy numbers of >10,000. Similarly, 1,552 and 1,684 known miRNAs were found in the LPS- and PBS-injected male samples, respectively (Fig 1B). Overall, there were fewer sRNAs in female ticks than those in male ticks.

**Fig 2:**
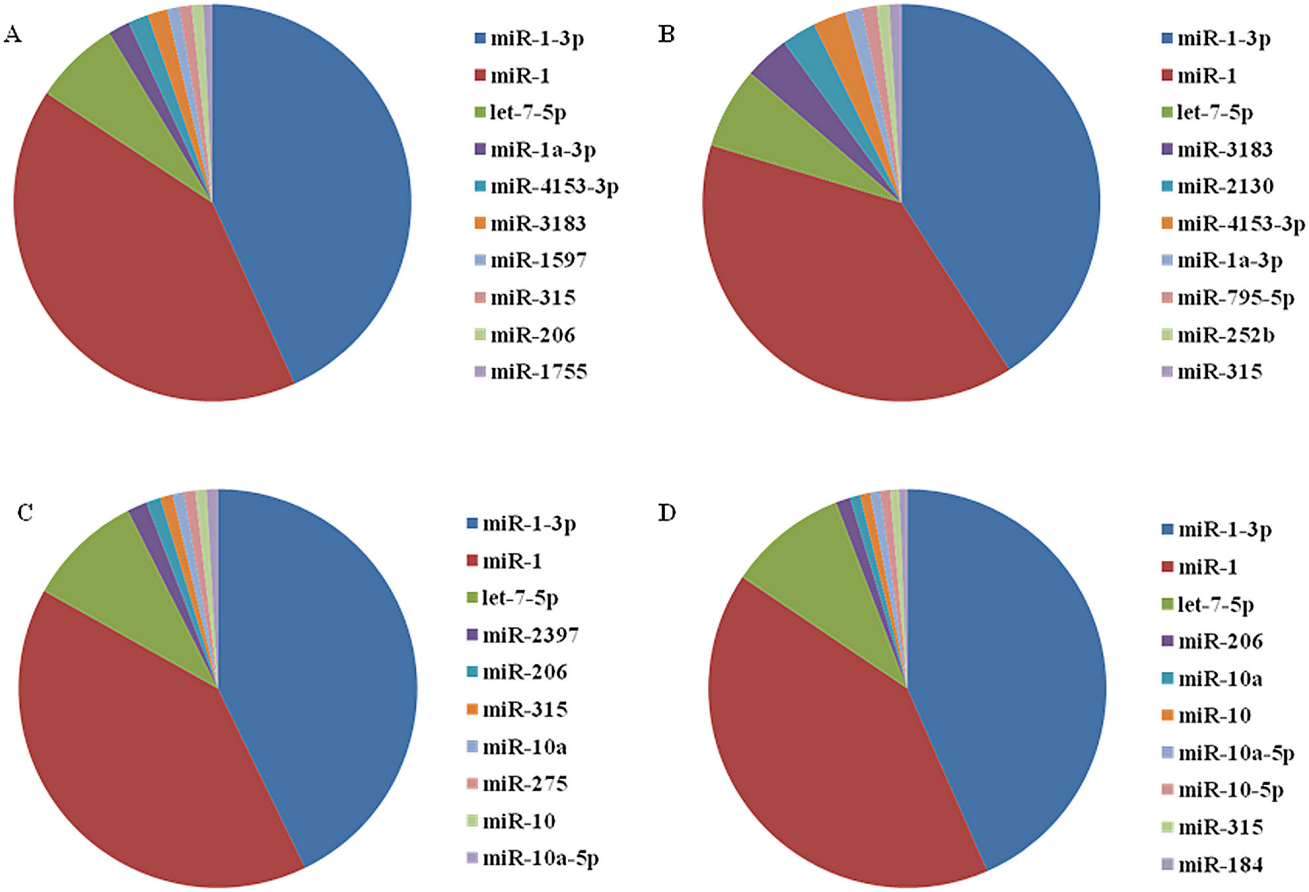
The ten most abundant miRNAs in the four samples. Panels A–D showed the percentage expression of the 10 most abundant miRNA for A) PBS-injected male samples, B) LPS-injected male samples, C) PBS-injected female samples, D) LPS-injected female samples.

Previous reports indicated that evolutionarily-conserved miRNAs are often highly expressed. BLAST searches in miRBase showed that 984 conserved miRNAs in LPS-injected female profiles belonged to 146 miRNA families, while the other miRNAs of the three tick samples all belonged to the same 267 families. This showed that the number of miRNA families decreased only for female ticks after LPS stimulation. The 10 top abundant miRNAs from the four samples are shown in Fig 2. miR-1-3p, miR-1 and let-7-5p were all highly expressed in the four samples, and the three miRNAs were expressed with the highest abundance. Thus, the highly expressed miRNAs may be evolutionarily-conserved miRNAs in *R*. *haemaphysaloides*. However, some of the 10 top abundant miRNAs also changed significantly with LPS induction in terms of miRNA families and the order of their abundance (S5 Table). For example, miR-2397 had only four copies in LPS-injected females, although it accounted for 1.33% (67,578) of the total miRNA abundance from PBS-injected females; miR-2130 and miR-795 were expressed in LPS-injected but not PBS-injected male ticks. Additionally, miR-1755 was expressed in male but not in female ticks, indicating a significant sexual difference. Four of the 10 most abundant miRNAs were highly expressed in the four samples: that is, miR-1-3p, miR-1, let-7-5p and miR-315. These conserved families are present in a large range of organisms, indicating that miRNAs from *R*. *haemaphysaloides* are distributed widely.

Based on miRBase version 21, 48 miRNAs in *R*. *haemaphysaloides* were highly consistent with thos of *I*. *scapularis* (S6 Table). All these miRNAs were expressed in the four samples, except for miR-307, miR-100, miR-5311 and miR-5313. Expression of miR-307 and miR-100 was low, and the miRNAs occurred in only two of the four samples. Additionally, some miRNAs showed no significant differences among the four tick samples, while some in female ticks were expressed more highly than those in male ticks such as miR-375, miR-275, miR-184, miR-10 and miR-1. The higher expression in the female ticks is likely related to many important functions such as innate immunity, blood feeding and development. Finally, expression of miR-252b in female ticks was lower than that in male ticks; and let-7 was the one highly expressed in LPS-induced female ticks.

### Identification of novel tick miRNAs

**Fig 3:**
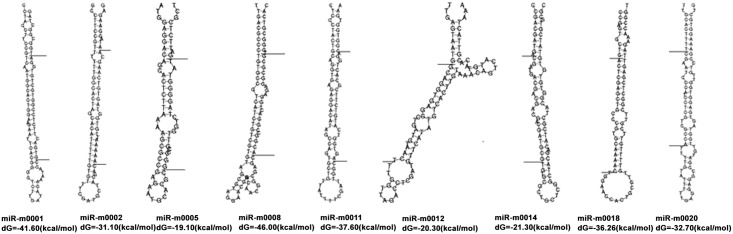
The putative precursor structures of the novel miRNAs in *R*. *haemaphysaloides*. The ends of mature miRNAs were marked with the lines.

We used prediction software miRDeep to determine whether non-annotated sequences mapped to the *I*. *scapularis* genome demonstrated folding properties of pre-miRNA hairpins. According to secondary structure, the dicer cleavage site and the minimum free energy, there were 10, 14, 18 and 20 novel miRNAs from profiles of PBS- and LPS-injected male and PBS- and LPS-injected female ticks, respectively. Information concerning the sequences, expression levels, minimal folding free energy and locations of the novel miRNA candidates are shown in S1–S4 Texts. We identified nine novel miRNAs present in at least two groups: five novel miRNAs were present in two of the four samples; two novel miRNAs in three samples; and female LPS-m0002_3p and female LPS-m0012_5p were in four samples. Female LPS-m0002_3p, male PBS-m0001_3p, female PBS-m0002_3p and male LPS-m0003_3p shared the same mature miRNAs and precursors (Table 6), although their expression levels differed. The putative precursor structures of the novel miRNAs are shown in Fig 3.

**Table 6:** Novel miRNAs identified in the four samples.

Female LPS^a^	miRNA	miRNA*	Sequence	Length	Female PBS^b^	Male LPS^c^	Male PBS^d^	Location^e^
m0001	35	0	GTGACTTCTCCGGTGCTGTGGA	22	m0001	N	N	3p
**m0002**	36	0	AAAAATTGTGGTAGTGTCAAGCA	23	m0002	m0003	m0001	3p
m0005	6	0	GGCCCGTTGGTCTAGGGGTAT	21	m0006	m0005	N	3p
m0008	8	0	ACGGCCGGACTGGAGAGGCGCG	22	N	m0008	N	3p
m0011	119	0	AGTGGTCATGTCTTCGCACTGGA	23	m0010	N	N	3p
**m0012**	11	0	GCAGGTGAGGCTGATGTAACT	21	m0011	m0010	m0006	5p
m0014	25	0	CGATGACGACGACGACGATGCG	22	N	m0013	N	5p
m0018	55	0	ACTCGAGCTGCCCGTGCAAAAC	22	m0017	N	m0010	5p
m0020	8	0	CGAATCCCATCCTCGTCGCCA	21	m0018	N	N	5p

^a^: Novel miRNA name in the LPS-injected female samples;

^b^: novel miRNA also in the PBS-injected female samples;

^c^: novel miRNA also in the LPS-injected male samples;

^d^: novel miRNA also in the PBS-injected male samples;

^e^: location of mature miRNA at the 5p or 3p arm of its precursor; N: no existence; miRNA

* means passenger strand.

### Differential expression of miRNAs between LPS- and PBS- treated female ticks

**Fig 4:**
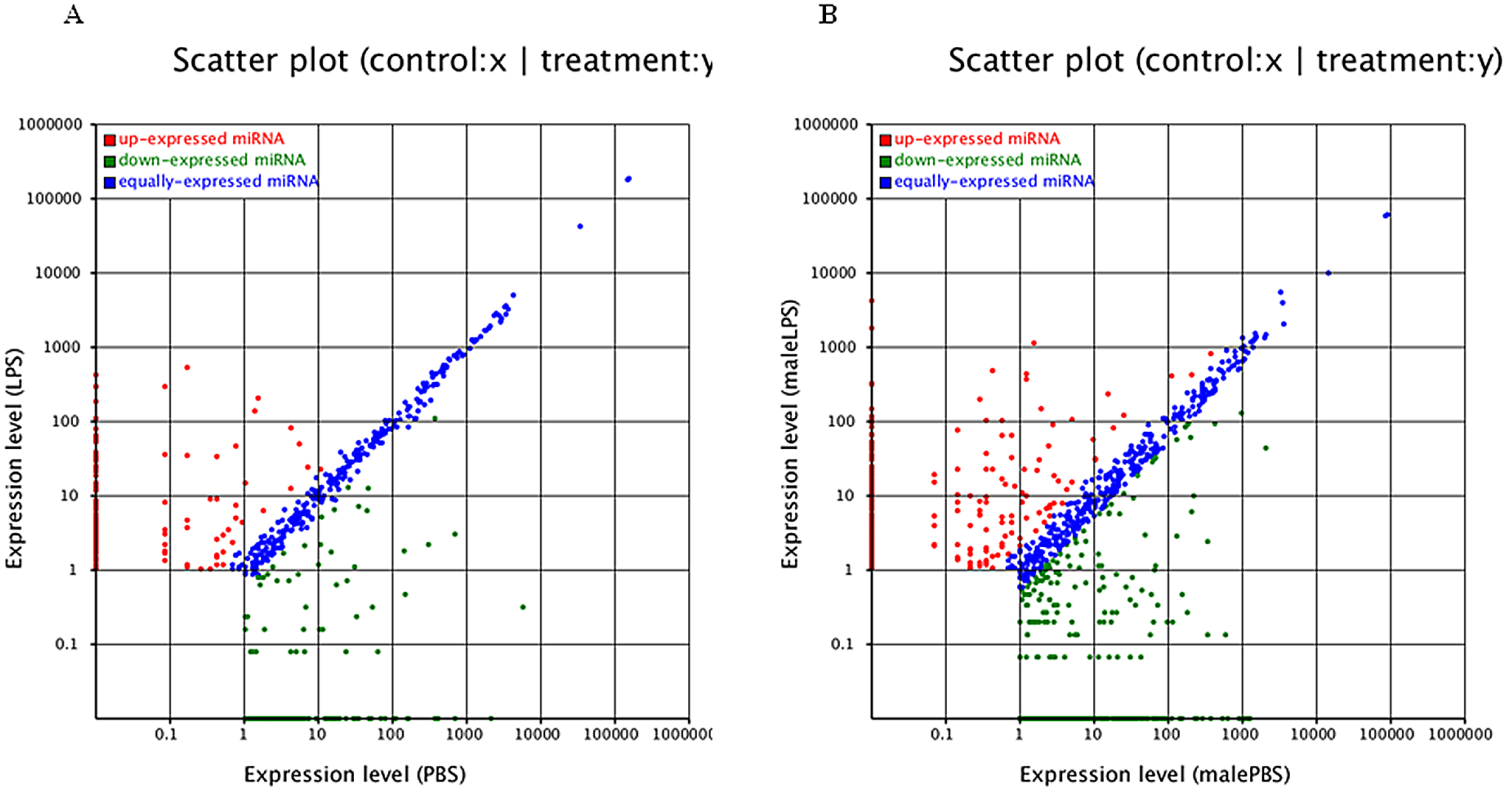
Scatter plot for differential expression analysis of miRNAs. A) Scatter plot for differential expression analysis of miRNAs between LPS- and PBS-injected female samples. B) Scatter plot for differential expression analysis of miRNAs between LPS- and PBS-injected male samples.

**Fig 5:**
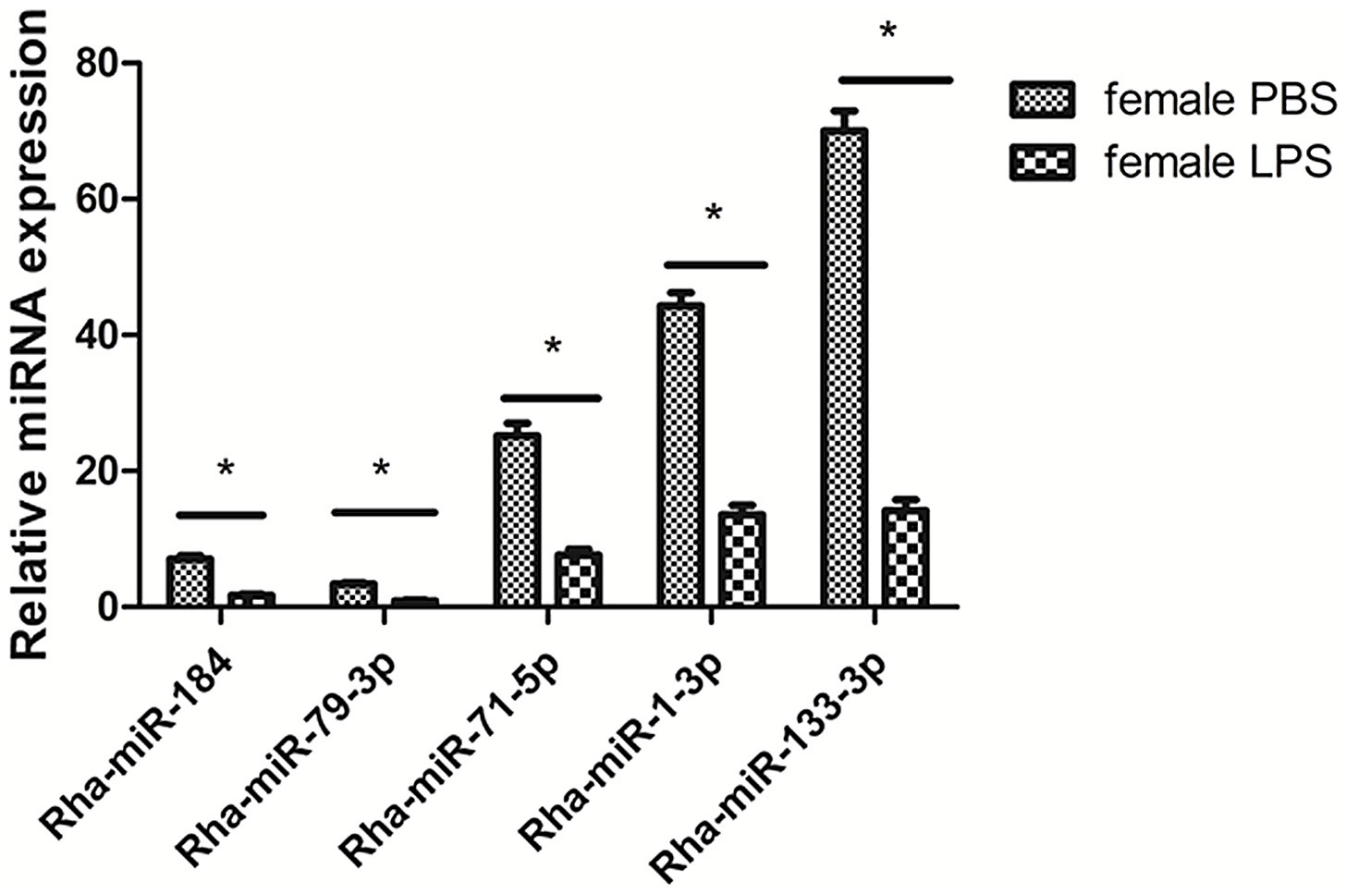
Investigation of the selected miRNAs in female ticks treated with PBS or LPS. * means P<0.01 by Student's *t*-test.

The global miRNA expression levels in samples were investigated by normalizing all known miRNA transcripts in each sample as reads per million. The fold-change and P-value were also calculated, leading to generation of a log_2_ ratio plot and a scatter plot (Fig 4A). We found that 350 miRNAs were expressed within twofold, of which 316 miRNAs were expressed within onefold in PBS- and LPS-injected female samples, including all 10 of the most abundant miRNAs from LPS-injected female samples. We also found that 150 known miRNAs were up-regulated after LPS stimulation (>2-fold), and of these, 37 showed >10-fold up-regulation (Table 7), with the highest increase for miR-357 (>15-fold). There were 160 known miRNAs down-regulated after LPS stimulation (>2-fold), of which 33 showed >10-fold down-regulation (Table 8). miR-3807-5p, miR-1287, miR-3152-3p and miR-466gshowed >15-fold decrease, compared to the PBS-injected female ticks. miR-2397, one of the 10 top abundant miRNAs in the PBS-injected female samples, was down-regulated 14-fold (S7 Table). Five miRNAs were selected for verification of miRNA expression and to confirm their down-regulation after LPS induction by qRT-PCR. As expected, the five selected miRNAs were all detected in PBS- and LPS-injected female ticks, and the down-regulation of the miRNAs was significant after LPS stimulation (Fig 5), consistent with the results of the high-through-put sequencing.

**Table 7:** miRNAs with up-regulated expression (>10-fold) after LPS induction in female ticks. miRNAs with >15-fold change are underlined. miRNA

bantam*	miR-2012	miR-357	miR-4271	miR-5453
miR-1000	miR-2064	miR-36b*	miR-4819*	miR-669b-5p
miR-1255a	miR-2072	miR-3863-3p	miR-497-5p	miR-81-3p
miR-1392	miR-2464-3p	miR-4000f-5p	miR-509-3-5p	miR-92e-5p
miR-1560*	miR-263b*	miR-4111-3p	miR-5365a	miR-961
miR-15a-3p	miR-303-5p	miR-4150-5p	miR-5435a	miR-98
miR-1653	miR-3102-5p.2-5p	miR-4211-5p	miR-4271	miR-99a-3p
miR-200b*	miR-3422	miR-4268	miR-4819*	

* indicates passenger strand, miR-5p or miR-3p means location of the mature miRNA at the 5p or 3p arm of its precursor.

**Table 8:** miRNAs with down-regulated expression (>10-fold) after LPS induction in female ticks. miRNAs with >15-fold change are underlined. miRNA.

miR-1287	miR-214	miR-2788-3p	miR-3850-5p	miR-5391
miR-1306	miR-2264	miR-2799	miR-3917	miR-540-3p
miR-152-3p	miR-2384	miR-309a-5p	miR-4008c-3p	miR-5614
miR-1682	miR-2397	miR-3139	miR-4140-3p	miR-64e
miR-1805	miR-2475	miR-3152-3p	miR-466g	miR-79-3p
miR-1805-3p	miR-2509	miR-3475	miR-4792	
miR-184*	miR-260	miR-3807-5p	miR-4861	

* indicates passenger strand, miR-5p or miR-3p means location of the mature miRNA at the 5p or 3p arm of its precursor.

### Differential expression of miRNAs between LPS- and PBS-treated male ticks

There were 528 miRNAs expressed within twofold, of which 491 were expressed within onefold change in PBS- and LPS-injected males (Fig 4B), including many of the 10 top abundant miRNAs from LPS-injected male ticks. There were 245 known miRNAs up-regulated in male ticks after LPS stimulation (>2-fold), of which 52 showed >10-fold up-regulation (Table 9). Compared with PBS-injected male ticks, mir-2130 and miR-795-5p were only expressed in LPS-injected male ticks, and increased by 15-fold (S8 Table).

**Table 9:** miRNAs with up-regulated expression (>10-fold) after LPS induction in male ticks. miRNAs with >15-fold change are underlined. miRNA.

miR-1227	miR-1905	miR-3785	miR-466g
miR-124-5p	miR-2130	miR-3865-5p	miR-4756-3p
miR-1329	miR-2220-3p	miR-4001d-5p	miR-4819*
miR-133-5p	miR-2226	miR-4139-3p	miR-4920
miR-1390	miR-2284i	miR-4154-3p	miR-5345b
miR-1419g*	miR-2429	miR-4158-5p	miR-669n
miR-149-3p	miR-2552	miR-4216-3p	miR-795-5p
miR-152-5p	miR-2754	miR-4217-3p	miR-798
miR-1591	miR-2788-3p	miR-4425	miR-80-3p
miR-1779	miR-2848	miR-4434	miR-84b
miR-1835	miR-3152-3p	miR-4526	miR-873-5p miR-942
miR-184-5p	miR-3180-3p	miR-4583	miR-m107-1-3p
miR-1890	miR-3585-3p	miR-460b	

* indicates passenger strand, miR-5p or miR-3p means location of the mature miRNA at the 5p or 3p arm of its precursor.

There were 354 known miRNAs down-regulated in male ticks after LPS stimulation (>2-fold), of which 59 had >11-fold down-regulation (Table 10). Eight miRNAs were down-regulated >15-fold. miR-1597, one of the 10 most abundant miRNAs, was down-regulated about fivefold in LPS-injected male compared with PBS-injected male ticks.

**Table 10:** miRNAs with down-regulated expression (>11-fold) after LPS induction in male ticks. miRNAs with >15-fold change are underlined. miRNA.

let-7f-1*	miR-279b-5p	miR-3807-5p	miR-4775
miR-1276	miR-2b-2-5p	miR-3814-3p	miR-4858
miR-133b-3p	miR-309a	miR-3823-5p	miR-520e
miR-1419g	miR-3121-3p	miR-392-5p	miR-548d-5p
miR-1553	miR-3150b-3p	miR-3967	miR-5553-3p
miR-1676	miR-3163	miR-4001g-3p	miR-677-5p
miR-1843	miR-3181	miR-4024-5p	miR-787-3p
miR-2032a	miR-3186-5p	miR-4169-3p	miR-80
miR-2152	miR-3201	miR-4179-3p	miR-84
miR-2228	miR-3339	miR-4195-3p	miR-92b-3p
miR-2469	miR-3567	miR-4220-5p	miR-93
miR-270	miR-36b*	miR-4544	miR-9-5p
miR-2705	miR-3717	miR-455	miR-963-3p
miR-2751	miR-3781	miR-455-3p	miR-98*
miR-2792-5p	miR-378f	miR-4625	

* indicates passenger strand, miR-5p or miR-3p means location of the mature miRNA at the 5p or 3p arm of its precursor.

**Fig 6:**
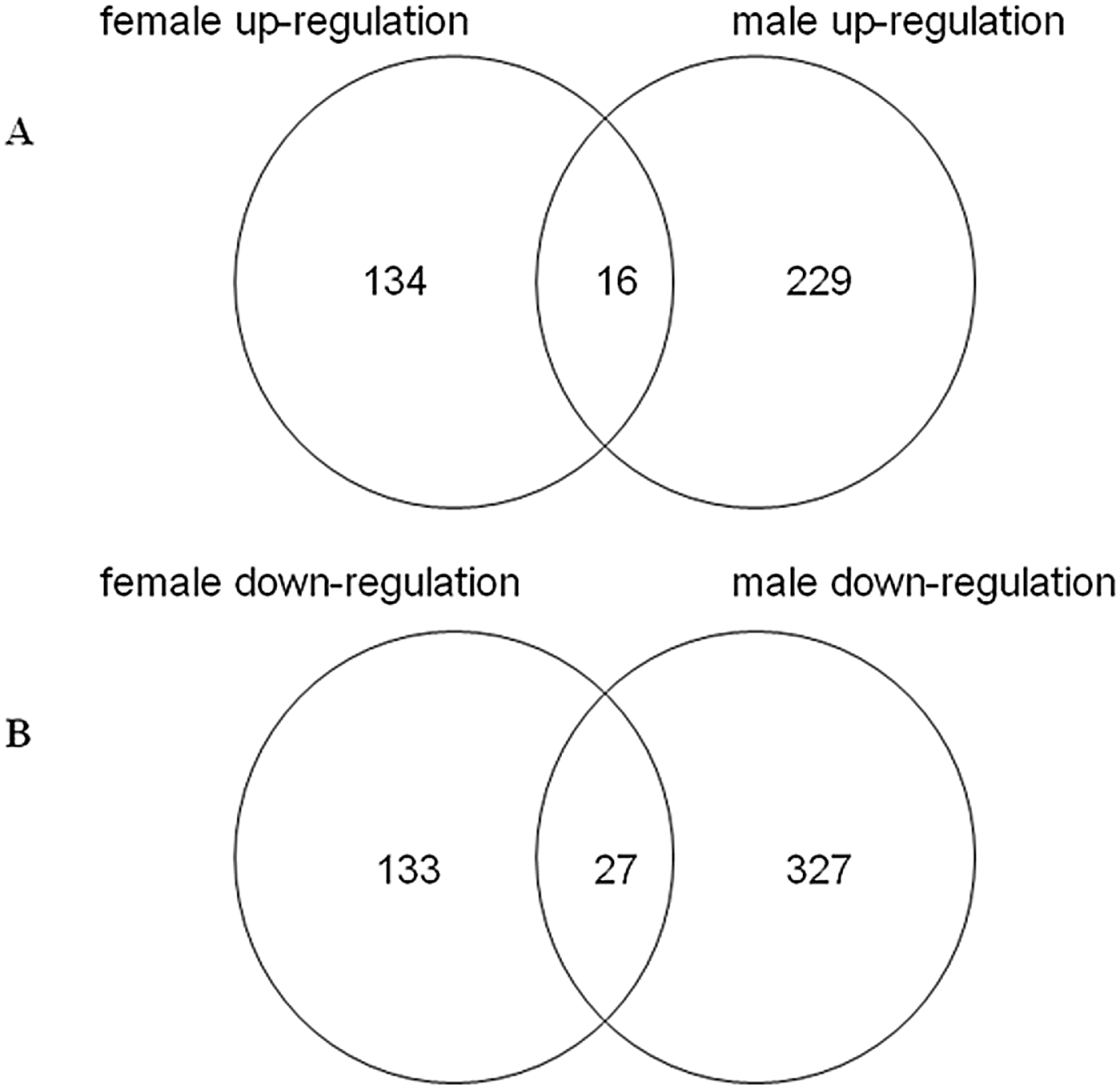
The miRNAs up-regulated >2-fold or down-regulated >2-fold in both male and female ticks after LPS injection. A, the miRNAs up-regulated >2-fold in female and male ticks after LPS injection; B, the miRNAs down-regulated >2-fold in female and male ticks after LPS injection.

In the female and male samples after LPS injection, the miRNAs up-regulated >2-fold or down-regulated >2-fold were analyzed. There were 16 miRNAs up-regulated in both female and male ticks after LPS stimulation, of these miR-2012, miR-2226, miR-2284i, miR-3422, miR-4150-5p and miR-4819* were up-regulated >10-fold (Fig 6A). Twenty-seven miRNAs were down-regulated >2-fold in both sexes after treatment (Fig 6B); of these, miR-1306, miR-2475, miR-309a-5p, miR-3807-5p and miR-184* were down-regulated >10-fold.

### Differential expression of miRNAs between female and male ticks

**Fig 7:**
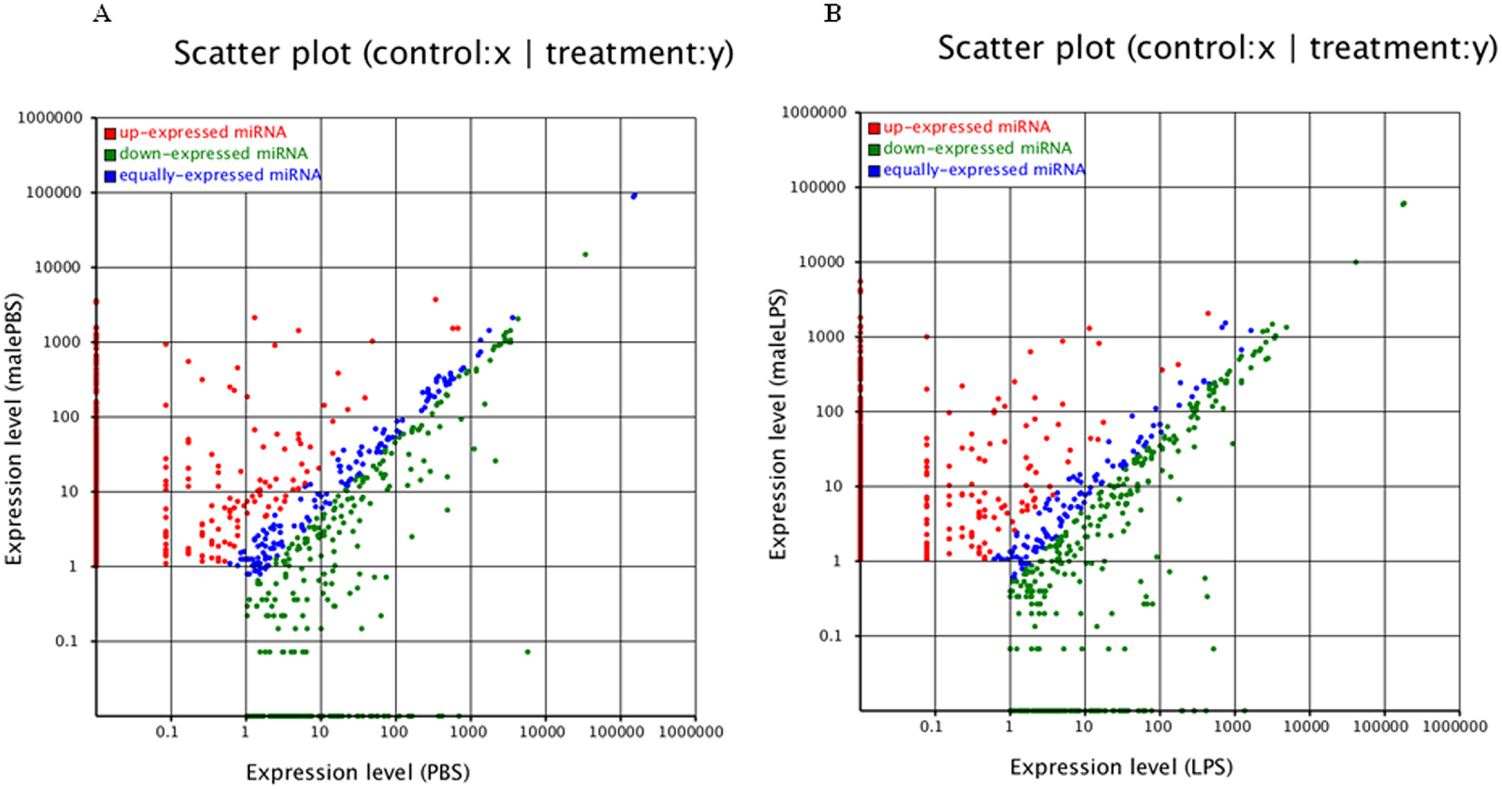
Scatter plot for differential expression analysis of miRNAs. A) Scatter plot for differential expression analysis of miRNAs between PBS-injected male and female samples. B) Scatter plot for differential expression analysis of miRNAs between LPS-injected male and female samples.

There were 224 miRNAs expressed in both male and female ticks, while the common miRNAs in the four samples included all 10 of the top abundant miRNAs in LPS-injected female ticks. For PBS-treated ticks (Fig 7A), 542 known miRNAs in male ticks showed higher expression than those in female ticks (>2-fold) (S9 Table). In addition, 83 known miRNAs in male ticks showed >12-fold up-regulation compared to female ticks (Table 11). However, 198 known miRNAs in male ticks showed lower expression than those in female ticks. There were 33 known miRNAs in the PBS-injected female samples that showed >10-fold down-regulation compared to male ticks (Table 12). In LPS-injected ticks (Fig 7B), 415 known miRNAs in male ticks were expressed more highly than those in female ticks (S10 Table). Moreover, 101 known miRNAs in male ticks showed up-regulation of >11-fold (Table 13), and almost all were not detected in female ticks, except for miR-196b-3p and miR-466i-5p. However, 220 known miRNAs in male ticks showed lower expression than those in female ticks (>2-fold), among which 41 were down-regulated >10-fold and were detected in the female ticks not in the male ticks, with the exceptions of miR-5606 and bantam* (Table 14).

**Table 11:** miRNAs with up-regulated expression (>12-fold) between PBS-injected female and male samples. miRNAs with >15-fold change are underlined. miRNA

let-7f-1*	miR-2690	miR-36	miR-4625
miR-1004-3p	miR-2696	miR-374b-3p	miR-4775
miR-1005-3p	miR-270	miR-3781	miR-4858
miR-124b*	miR-2705	miR-378b	miR-4870
miR-126	miR-2788	miR-378f	miR-4942-3p
miR-1419g	miR-2789	miR-3823-5p	miR-4962-3p
miR-1653	miR-3002	miR-3836a-3p	miR-509
miR-1669	miR-3004	miR-392-5p	miR-520e
miR-1676	miR-302*	miR-4000f-5p	miR-548ap-5p
miR-1755	miR-3070b-3p	miR-4001g-3p	miR-548d-5p
miR-1842	miR-3113-3p	miR-4024-5p	miR-553
miR-200b	miR-3121-3p	miR-4071-5p	miR-664-1*
miR-2032a	miR-3163	miR-4110-5p	miR-669e-5p
miR-2228	miR-3181	miR-4153-3p	miR-676
miR-2284p	miR-3183	miR-4268	miR-677-5p
miR-2284u	miR-3339	miR-430i	miR-694
miR-2304	miR-341	miR-43a*	miR-787-3p
miR-2496-5p	miR-3485-3p	miR-455	miR-93
miR-2513b	miR-3490	miR-455-3p	miR-963-3p
miR-2522b	miR-352	miR-459	miR-98*
miR-2575	miR-3567	miR-4617	

* indicates passenger strand, miR-5p or miR-3p means location of the mature miRNA at the 5p or 3p arm of its precursor.

**Table 12:** miRNAs with down-regulated expression (>10-fold) between PBS-injected female and male samples. miRNAs with >15-fold change are underlined. miRNA.

miR-1286	miR-1780	miR-2509	miR-3475	miR-466n-3p
miR-1287	miR-1805-3p	miR-2557*	miR-3850-5p	miR-5358b*
miR-1497a	miR-2047*	miR-260	miR-4006c-5p	miR-540-3p
miR-152-3p	miR-2264	miR-2788-3p	miR-4008c-3p	miR-64e
miR-152-5p	miR-2384	miR-2799	miR-4487	miR-738
miR-152a	miR-2397	miR-3139	miR-44a*	
miR-1682	miR-2399	miR-3152-3p	miR-466g	

* indicates passenger strand, miR-5p or miR-3p means location of the mature miRNA at the 5p or 3p arm of its precursor.

**Table 13:** miRNAs with up-regulated expression (>11-fold) between LPS-injected female and male samples. miRNAs with >15-fold change are underlined. miRNA

miR-1004-3p	miR-1b-3p	miR-3015a	miR-4067-5p	miR-4617
miR-1005-3p	miR-200a	miR-3070b-3p	miR-4071-5p	miR-466g
miR-1227	miR-2130	miR-3113-3p	miR-4110-5p	miR-466i-5p
miR-124b *	miR-214	miR-3152-3p	miR-4139-3p	miR-4920
miR-126	miR-214-3p	miR-3183	miR-4153-3p	miR-493-5p
miR-133-5p	miR-2220-3p	miR-341	miR-4154-3p	miR-4962-3p
miR-1375	miR-2365	miR-3490	miR-4158-5p	miR-509
miR-1382	miR-2414	miR-3585-3p	miR-4182-3p	miR-548aj-3p
miR-1421ai*	miR-2450a	miR-36	miR-4216-3p	miR-669e-5p
miR-149-3p	miR-2495-5p	miR-3674	miR-4217-3p	miR-676
miR-1591	miR-2496-5p	miR-369	miR-429*	miR-694
miR-1669	miR-2513b	miR-374b-3p	miR-430i	miR-795-5p
miR-1746	miR-2522b	miR-3785	miR-43a*	miR-80-3p
miR-1755	miR-2552	miR-379-5p	miR-4425	miR-86*
miR-1779	miR-2561	miR-3808-3p	miR-4438	miR-880-3p
miR-1835	miR-2575	miR-3836a-3p	miR-4499	miR-942
miR-1842	miR-2696	miR-3865-5p	miR-449a	miR-m107-1-3p
miR-184-5p	miR-2789	miR-3879-5p	miR-4526	
miR-1890	miR-2e-5p	miR-3917	miR-4583	
miR-1905	miR-3002	miR-4006b-5p	miR-459	
miR-196b-3p	miR-3004	miR-4054-5p	miR-460b	

* indicates passenger strand, miR-5p or miR-3p means location of the mature miRNA at the 5p or 3p arm of its precursor.

**Table 14:** miRNAs with down-regulated expression (>10-fold) between LPS-injected female and male samples. miRNAs with >15-fold change are underlined. miRNA

bantam*	miR-2464-3p	miR-4271	miR-64c
miR-1000	miR-263b*	miR-4327	miR-669b-5p
miR-1392	miR-3102-5p.2-5p	miR-4451	miR-81-3p
miR-13b-2-5p	miR-3201	miR-466n-3p	miR-84
miR-1560*	miR-357	miR-497-5p	miR-881
miR-15a-3p	miR-36b*	miR-509-3-5p	miR-92e-5p
miR-1780	miR-3863-3p	miR-5313	miR-961
miR-2047*	miR-4006c-5p	miR-5365a	miR-98
miR-2064	miR-4036-5p	miR-5435a	
miR-2072	miR-4051-3p	miR-5453	
miR-2211-3p	miR-4111-3p	miR-5606	

* indicates passenger strand, miR-5p or miR-3p means location of the mature miRNA at the 5p or 3p arm of its precursor.

## Discussion

In the present study, miRNAs were investigated for the first time in LPS- and PBS-injected female and male *R*. *haemaphysaloides* ticks. This fills in gaps in the information on miRNA profiles of tick species other than *H*. *longicornis* [16], *R*. *microplus* [15] and recently reported *Hyalomma anatolicum anatolicum* [22]. The miRNA profiles in female ticks were composed of two peaks including fragments of 20–22 and 26–28 bp (Fig 1A), consistent with previous results for *H*. *longicornis* [16] as well as female ticks of *Hyalomma*. *anatolicum anatolicum* [22]. Meanwhile, the size peak of miRNAs in male ticks was in the range of 22–26 bp (Fig 1A), which greatly differed from that of female ticks and may be explained by their different duties in breeding. The appearance of miR-275 in the 10 top abundant miRNAs (Fig 2C) of female but not of male ticks suggests its critical role in the former. It is also reported that miR-275 is indispensable for blood digestion and egg development in mosquitoes [23]. However, injection of LPS led to decrease in miR-275 and miR-2379, indicating changes in miRNA composition may be related to the innate immune system. The reduction of miR-275 indicates its relationship with innate immunity of ticks in addition to its functions identified above. These conserved miRNAs may have essential roles in ticks; however, further work is required in this field.

Several of the most abundantly expressed miRNAs in the LPS- and PBS-induced female samples are expressed in humans, suggesting a conserved functional role for these miRNAs throughout evolution. miR-184 regulates the expression of NFAT1 (nuclear factor of activated T cells-1) protein in umbilical cord blood CD4^+^ T cells [24]; miR-1 is involved in the clathrin pathway in the regulation of phagocytosis in shrimp, suggesting that miR-1 shares similar functions in the phagocytosis of shrimp hemocytes and mammalian macrophages [25]. In *D*. *melanogaster*, miR-1 is predicted to be involved in the regulation of Toll-like receptor signaling pathways [7]. Expression of miR-133b and miR-206 in the ll17a/f locus is co-regulated with IL-17 production in αβ and γδ T cells in humans, indicating that expression of miR-133b and miR-206 in T cells participates in Th17-type immune reactions [26]. In addition, miR-1-3p, miR-1 and let-7-5p represented the predominant proportion of the miRNAs in *R*. *haemaphysaloides*, regardless of the sex of ticks and injection of LPS (Fig 2); while in unfed *H*. *longicornis*, miR-1, miR-375 and miR-184 were the most abundant [16], and similarly for miR-1, let-7 and miR-184 in *R*. *microplus* [15]. This suggests an indispensable role of miR-1 for all tick species but the other two miRNAs may be responsible for species specificity. Moreover, nine novel miRNAs were detected in *R*. *haemaphysaloides* by deep sequencing and reference to the *I*. *scapularis* genome, and this will be tested in future work.

LPS was used to stimulate the innate immune system so that the changes in miRNA associated with the respective innate immunity of female and male ticks could be investigated. Injection of LPS led to higher expression of defensin in the tick *H*. *longicornis* [6]. Defensins as well as lectins, lysozymes, hebraein, microplusin and ixodidin make up the innate immunity of ticks [4]. As important regulators of both adaptive and innate immunity [27], miRNAs probably control the expression of these immune molecules directly or through Toll-like receptors. In the present study, the induction of LPS led to an increase in 37 miRNAs by >10-fold in female ticks, including miR-303-5p (Table 7). In *D*. *melanogaster*, miR-303-5p (with a previous ID as miR-303) is predicted to target pll (CG5974, a gene from the Toll pathway) and upd (CG5993, a gene associated with the JAK/STAT pathway) [7]. The significant up-regulation of miR-303 in LPS-treated female ticks hints at a similar function to that in *Drosophila*. Meanwhile, 33 miRNAs including miR-184*, miR-79-3p and miR-214 were down-regulated >10-fold in the female ticks after induction of LPS (Table 8). Dme-miR-184*, also called dme-miR-184-5p has putative targets including Dro (CG10816, a member of Imd pathway), Jra (CG2275) and kay (CG33956, members of JNK pathway) [7]. miR-79-3p (with a previous ID: miR-79) is assumed to regulate Pvr (CG8222) and puc (CG7850), two members of the JNK pathway [7]. miR-214 secreted by cancer cells enhances immune suppression and tumor implantation/growth in mice [28], although whether and how it regulates innate immunity in arthropods remains unknown.

In LPS-treated male ticks, 52 miRNAs were up-regulated >10-fold after LPS injection, including miR-124-5p (Table 9), which was predicted to regulate the genes of the Imd and JNK pathways in *D*. *melanogaster* [7]. Alternatively, 59 down-regulated miRNAs decreased by >11-fold were detected in LPS-injected male ticks (Table 10), including miR-2b, miR-9-5p and miR-279b which were assumed to regulate one or two immune pathways in *D*. *melanogaster* [7]. A previous study indicated that miR-9 played an important role in the anti-inflammatory regulation of LPS-activated microglia cells by molecular hydrogen [29]. Consistent with this, miR-9-5p in LPS-injected male ticks was down-regulated about 12-fold in the present study, indicating a similar function of miR-9 in innate immunity of male ticks. However, in female ticks, the decrease in miR-9 induced by LPS was less obvious than that in male ticks. The reason is unknown but we speculate that male ticks, as a mating guard for females during feeding, undertake the duty of excluding the exogenous threat as well as attack by host antibodies [30]. In addition, Freitak investigated the miRNAs involved in the immune responses by injection of *Tribolium castaneum* (red flour beetle) with peptidoglycan, a reagent with high capacity to induce immune reaction, and found that 59 miRNAs were differentially expressed, which also included miR-79 and miR-124 [31]. This supports our opinion that miR-79 and miR-124 are involved in regulation of innate immunity. All these hypotheses will be studied and described in the future.

It is reported that conserved miR-8 decreases with infection, which regulates innate immune homeostasis by targeting Toll and Dorsal in *Drosophila* [8,9]. In *P*. *xylostella*, miR-8 regulates activation of the Toll pathway and prophenoloxidase by targeting Serpin 27 [10]. However, we did not find any differential expression of miR-8 in LPS-induced ticks (data not shown), which may be associated with species specificity of the ticks.

In beetles, only 11 miRNAs showed differential expression between females and males [31]. In the present study, 542 miRNAs showed higher expression and 198 miRNAs showed lower expression in PBS-injected male ticks. This discrepancy between ticks and beetles suggests evolution in female ticks of a more complicated regulatory system for sucking a large amount of blood from hosts and subsequent reproduction.

## Conclusions

Four sRNA libraries from the LPS- and PBS-injected female and male *R*. *haemaphysaloides* ticks were generated. There was a greater abundance of specific LPS-induced female sRNAs, compared to the specific PBS-injected female sRNAs in terms of both unique and total sRNAs. Eight of the 10 top abundant miRNAs were detected in both the PBS- and LPS-injected female ticks: that is, miR-1-3p, miR-1, let-7-5p, miR-206, miR-10a, miR-10, miR-10a-5p and miR-315. There were 350 miRNAs expressed within twofold in the PBS- and LPS-injected female ticks and 150 known miRNAs were up-regulated, with the highest increase for miR-357 (>15-fold); while 160 known miRNAs were down-regulated with the most significant down-regulation of four miRNAs: miR-3807-5p, miR-1287, miR-3152-3p and miR-466g (>15-fold). Seven of the 10 top abundant miRNA were found in both the LPS- and PBS-induced male ticks: that is, miR-1-3p, miR-1, let-7-5p, miR-1a-3p, miR-4153-3p, miR-3183 and miR-315. After LPS injection of male ticks, 528 miRNAs were expressed within twofold compared to the PBS-treated group, and 245 known miRNAs were up-regulated >2-fold, with the highest expression of mir-2130 and miR-795-5p (>15-fold). Meanwhile, 354 known miRNAs were down-regulated >2-fold. The eight most down-regulated miRNAs were miR-4858, miR-2032a, miR-787-3p, miR-93, miR-3121-3p, miR-963-3p, miR-548d-5p and miR-378f (>15-fold). This work provides the first information on the relationship between miRNAs and innate immunity of ticks and establishes a platform for further study.

## Supporting Information

S1 Table sRNAs of matching known miRNAs in PBS-injected female ticks. (XLSX)

S2 Table sRNAs of matching known miRNAs in LPS-injected female ticks. (XLSX)

S3 Table sRNAs of matching known miRNAs in PBS-injected male ticks. (XLSX)

S4 Table sRNAs of matching known miRNAs in LPS-injected male ticks. (XLSX)

S5 Table Summary of known miRNAs in the four tick samples. (XLSX)

S6 Table Comparison of miRNAs between the four tick samples and *I*. *scapularis* miRBase. (XLSX)

S7 Table Comparison of changes between LPS- and PBS-injected female samples. (XLSX)

S8 Table Comparison of changes between LPS- and PBS-injected male samples. (XLSX)

S9 Table Comparison of changes between PBS-injected female and male samples. (XLSX)

S10 Table Comparison of changes between LPS-injected female and male samples. (XLSX)

S1 Text Details of the novel miRNAs and precursors of the PBS-injected male samples. (PDF)

S2 Text Details of the novel miRNAs and precursors of the LPS-injected male samples. (PDF)

S3 Text Details of the novel miRNAs and precursors of the PBS-injected female samples. (PDF)

S4 Text Details of the novel miRNAs and precursors of the LPS-injected female samples. (PDF)
