# Supplementary material for: Lipopolysaccharide-Induced Differential Expression of miRNAs in Male and Female Rhipicephalus haemaphysaloides Ticks
Source: PLoS One. 2015 Oct 2;10(10):e0139241. doi: 10.1371/journal.pone.0139241 (PMC4592253; doi:10.1371/journal.pone.0139241)
Supplement: S1 Text — (PDF) [file pone.0139241.s011.pdf]

mireap
malePBS-m0001 DS633978:113214:113295:+ 82(nt) -31.10(kcal/mol)
GCCTTCCGTTTTTTGGCACTAGCACATTTTTGTGTTTCGATGCTACGACAAAAATTGTGGTAGTGTCAAGCAATAGGAAGAG
malePBS-m0001 18
..((((((((.((((((((((.(((((((((((((.........)).))))))).)))).)))))))))).))).)))))..
AAAAATTGTGGTAGTGTCAAGCA*******
malePBS-m0001-3p 18
--CAAAAATTGTGGTAGTGTCAA-------------
t1124477 1
--CAAAAATTGTGGTAGTGTCAAGC-----------
t1386551 1
--CAAAAATTGTGGTAGTGTCAAGCA----------
t0277923 2
---AAAAATTGTGGTAGTGTCAAG------------
t0330849 2
---AAAAATTGTGGTAGTGTCAAGC-----------
t0286540 2
---AAAAATTGTGGTAGTGTCAAGCA----------
t0074040 10
//
mireap
malePBS-m0002 DS682300:151460:151546:- 87(nt) -37.30(kcal/mol)
TTGCCGACCTATGTTGATCGGGTGTCCGCGCTATGCTGGGCGTCGATATGGTGACCCCAGGCGACCCCGGGGCGACCAGGTCGCCTA
malePBS-m0002 12
..(.((((((..((((.(((((.(((.((......(((((.((((......)))))))))))))))))))..)))).)))))).)..
**********ATGTTGATCGGGTGTCCGCGC**
malePBS-m0002-5p 10
---------TATGTTGATCGGGTGTCC--
t1734433 1
----------ATGTTGATCGGGTGTCCGCGC--
t0289222 2
-----------TGTTGATCGGGTGTCCGC--
t1896961 1
-----------TGTTGATCGGGTGTCCGCGC--
t2038372 1
-----------TGTTGATCGGGTGTCCGCGCT---
t1965664 1
-----------TGTTGATCGGGTGTCCGCGCTA--
t0768483 1
-----------TGTTGATCGGGTGTCCGCGCTAT---
t1746811 1
------------GTTGATCGGGTGTCCGCGC--
t0777568 1
------------GTTGATCGGGTGTCCGCGCT---
t1263754 1

-----------------------------------CTGGGCGTCGATATGGTGACCC------------------------------
t1706723 1
--CGTCGATATGGTGACCCCAGG--------------------------
t1784950 1
//
mireap
malePBS-m0003 DS762741:4587:4681:+ 95(nt) -18.60(kcal/mol)
ACGCATCATGATCCTCATGCTGCTGTCTCTTATGATGATTTTGAGTAGGACTATGATGATAATGACGATGACGACGACGACGATGATGAT
ACGCG malePBS-m0003 8
.((((((((.(((.((....((.((((((((((.((.(((...((.....))..))).)).)))).)).)))).))..)).))).)))))
..)))
GACGATGACGACGACGACGATG**
***** malePBS-m0003-3p 8
---GACGATGACGACGACGACGATG-----
----- t0207484 3
---CGATGACGACGACGACGAT------
----- t0423724 2
---CGATGACGACGACGACGATG-----
----- t0219827 3
//
mireap
malePBS-m0004 DS767867:1050:1149:+ 100(nt) -30.70(kcal/mol)
TCTGTGTCGACGAGGACACCGCCATGCTGCAGACCGTGACAACACACAAGGGCCTGTTCAAGGTGACGCGATTGCAGTTTGGTGTGGCAG
TGGCAGTAGC malePBS-m0004 5
.(((((((.((...(.(((.((((.(((((((.((((.((.....(((......))).....)).))).).))))))).))))))).).)
))))).))).
**CGCGATTGCAGTTTGGTGTGGCAG
********** malePBS-m0004-3p 5
--CGCGATTGCAGTTTGGTGT-----
---------- t1079932 1
--CGCGATTGCAGTTTGGTGTG----
---------- t1318803 1
--CGCGATTGCAGTTTGGTGTGG---
---------- t1913237 1
--CGCGATTGCAGTTTGGTGTGGC--
---------- t1643464 1

CGCGATTGCAGTTTGGTGTGGCAG---------- t1068335 1
//
mireap
malePBS-m0005 DS790259:165907:165994:- 88(nt) -22.70(kcal/mol)
GCGGTGCCCTCTTCAGCCGTCCGGTCGGCGTCTGCTCATGGCTGCCGCTTGCCCTCCATTCAGTGTAGAGCCTTCGGAATTCAACCTT
malePBS-m0005 9
(((((..........)))))((((..(((.(((((.(((((..((.....))...))))...).)))))))).))))...........
TCCATTCAGTGTAGAGCCTTCGGA********

malePBS-m0005-3p 8
----TGCCCTCTTCAGCCGTCCGGTCG---
t2031559 1
--TCCATTCAGTGTAGAGCCTTCGG-----------
t0405969 2
--TCCATTCAGTGTAGAGCCTTCGGA----------
t0161680 4
--TCCATTCAGTGTAGAGCCTTCGGAA---------
t1915371 1
--TCCATTCAGTGTAGAGCCTTCGGAAT--------
t1803948 1
//
mireap
malePBS-m0006 DS845963:700:788:+ 89(nt) -20.30(kcal/mol)
TTGAGTAATGGCAGGTGAGGCTGATGTAACTTTGGTTAGACCTCGATGTTCATATGTCACTTGTAAACAGTCATGTACACGTTACTAAA
malePBS-m0006 10
...(((((((((((((((.(...(((.(((...((.....)).....)))))).).))))))))..(((....)))...)))))))...
**********GCAGGTGAGGCTGATGTAACT**
malePBS-m0006-5p 10
--------TGGCAGGTGAGGCTGATGTAACTT---
t2013174 1
----------GCAGGTGAGGCTGATGTAACT--
t0096061 7
----------GCAGGTGAGGCTGATGTAACTT---
t0310786 2
//
mireap
malePBS-m0007 DS874548:52973:53057:+ 85(nt) -30.10(kcal/mol)
TACCTCACCTGAGCTGGAAGAGTTAGATCTGGCACCACCGACAGCGCTCAAGTGTCAGGATCCCACTTCCAACTCGAGTGGGACG
malePBS-m0007 6
...(((((.((((.((((((.(...((((((((((...((....)).....)))))).)))).).)))))).)))).)))))...
**********GAGCTGGAAGAGTTAGATCT***
malePBS-m0007-5p 6
----------GAGCTGGAAGAGTTAGATCT---
t0108978 6
//
mireap
malePBS-m0008 DS941638:154652:154714:+ 63(nt) -26.00(kcal/mol)
TGCTTTACTGTCCTGTTCTCGGCACGACGCCTTCCCGTGCCGGTGCCGAAACGGGGTGAAGAC malePBS-m0008 7
..((((((...((((((.((((((((.(((......))).).)))))))))))))))))))..
**********TCCTGTTCTCGGCACGACGC********************************* malePBS-m0008-5p 7
----------TCCTGTTCTCGGCACGACG---------------------------------- t0367738 2
----------TCCTGTTCTCGGCACGACGC--------------------------------- t0209905 3
----------TCCTGTTCTCGGCACGACGCCT------------------------------- t0755238 1
-----------CCTGTTCTCGGCACGACG---------------------------------- t1907684 1

```
//
mireap
malePBS-m0009 DS945001:228263:228349:- 87(nt) -39.40(kcal/mol)
AGTCACTAGTTAATTGTACTTCATCAGGTGCTCTGGAGTTCGGTTCGCACAACCAGGCATCTTTTGGAGTGCAAATGATAAGTGGCA
malePBS-m0009 11
.((((((.((((.((((((((((..((((((.((((.((.((...)).))..))))))))))..)))))))))).)))).)))))).
******************************************************AGGCATCTTTTGGAGTGCAAATG**********
malePBS-m0009-3p 11
-----------------------------------------------------CAGGCATCTTTTGGAGTG----------------
t0727296 1
-----------------------------------------------------CAGGCATCTTTTGGAGTGCAAATG----------
t0223350 3
------------------------------------------------------AGGCATCTTTTGGAGTGCAAA------------
t1120048 1
------------------------------------------------------AGGCATCTTTTGGAGTGCAAATG----------
t0119818 6
//
mireap
malePBS-m0010 DS966815:32756:32835:- 80(nt) -36.26(kcal/mol)
CCGAGTGCTGACTCGAGCTGCCCGTGCAAAACTGGAACCACTGCGTTGTTTGTTCGTTCGGCTCGAGTTAGAAACCGGT malePBS-
m0010 21
(((.((.((((((((((((..((.(((((((..............))))))).))..))))))))))))..))))).
**********ACTCGAGCTGCCCGTGCAAAAC************************************************ malePBS-
m0010-5p 21
----------ACTCGAGCTGCCCGTGCAA--------------------------------------------------- t0401585
2
----------ACTCGAGCTGCCCGTGCAAA-------------------------------------------------- t0217735
3
----------ACTCGAGCTGCCCGTGCAAAA------------------------------------------------- t0172878
4
----------ACTCGAGCTGCCCGTGCAAAAC------------------------------------------------ t0084495
8
----------ACTCGAGCTGCCCGTGCAAAACT----------------------------------------------- t0159198
4
//
```
