# Supplementary material for: Lipopolysaccharide-Induced Differential Expression of miRNAs in Male and Female Rhipicephalus haemaphysaloides Ticks
Source: PLoS One. 2015 Oct 2;10(10):e0139241. doi: 10.1371/journal.pone.0139241 (PMC4592253; doi:10.1371/journal.pone.0139241)
Supplement: S2 Text — (PDF) [file pone.0139241.s012.pdf]

mireap
maleLPS-m0001 DS612599:38782:38860:- 79(nt) -34.40(kcal/mol)
CCAGACAAAACTGGTTTTCACAATGATCGTCCAGATGGACCTGAAGTCTGAGATCATTGTGAAAGCTGATTTTGTTGAT maleLPS-m0001 4069
...(((((((..((((((((((((((((...(((((.........))))).))))))))))))))))..)))))))...
**********CTGGTTTTCACAATGATCGTCCA** maleLPS-m0001-5p 4066
--------AACTGGTTTTCACAATGATCGTCCA-- t2356262 1
--------AACTGGTTTTCACAATGATCGTCCAG--- t2297092 1
---------ACTGGTTTTCACAATGATCGTCC--- t1769754 1
---------ACTGGTTTTCACAATGATCGTCCAG--- t0265620 3
---------ACTGGTTTTCACAATGATCGTCCAGA-- t2358968 1
----------CTGGTTTTCACAATGATCGT--- t0017902 62
----------CTGGTTTTCACAATGATCGTC-- t0007384 174
----------CTGGTTTTCACAATGATCGTCC--- t0011653 102
----------CTGGTTTTCACAATGATCGTCCA-- t0000653 2352
----------CTGGTTTTCACAATGATCGTCCAG--- t0001408 1098
----------CTGGTTTTCACAATGATCGTCCAGA-- t0007672 166
----------CTGGTTTTCACAATGATCGTCCAGAT--- t0142256 6
-----------TGGTTTTCACAATGATCGTC-- t1053426 1
-----------TGGTTTTCACAATGATCGTCC--- t0816411 1
-----------TGGTTTTCACAATGATCGTCCA-- t0023829 45
-----------TGGTTTTCACAATGATCGTCCAG--- t0037108 27
-----------TGGTTTTCACAATGATCGTCCAGA-- t0093179 9
------------GGTTTTCACAATGATCGTCC--- t1874106 1
------------GGTTTTCACAATGATCGTCCA-- t0962557 1
------------GGTTTTCACAATGATCGTCCAG--- t0081904 11
------------GGTTTTCACAATGATCGTCCAGA-- t0238880 3
---------------TTTCACAATGATCGTCCA-- t0907488 1
---------------TTTCACAATGATCGTCCAG--- t2000047 1
---------------TTTCACAATGATCGTCCAGA-- t1637467 1
//
mireap
maleLPS-m0002 DS616556:1188:1266:- 79(nt) -34.40(kcal/mol)
CCAGACAAAACTGGTTTTCACAATGATCGTCCAGATGGACCTGAAGTCTGAGATCATTGTGAAAGCTGATTTTGTTGAT maleLPS-m0002 4069

...((((((..((((((((((((((((...(((((.........))))).)))))))))))))))))..)))))))...
**********CTGGTTTTCACAATGATCGTCCA** maleLPS-m0002-5p 4066
--------AACTGGTTTTCACAATGATCGTCCA--- t2356262 1
--------AACTGGTTTTCACAATGATCGTCCAG-- t2297092 1
---------ACTGGTTTTCACAATGATCGTCC-- t1769754 1
---------ACTGGTTTTCACAATGATCGTCCAG-- t0265620 3
---------ACTGGTTTTCACAATGATCGTCCAGA--- t2358968 1
----------CTGGTTTTCACAATGATCGT-- t0017902 62
----------CTGGTTTTCACAATGATCGTC--- t0007384 174
----------CTGGTTTTCACAATGATCGTCC-- t0011653 102
----------CTGGTTTTCACAATGATCGTCCA--- t0000653 2352
----------CTGGTTTTCACAATGATCGTCCAG-- t0001408 1098
----------CTGGTTTTCACAATGATCGTCCAGA--- t0007672 166
----------CTGGTTTTCACAATGATCGTCCAGAT-- t0142256 6
-----------TGGTTTTCACAATGATCGTC--- t1053426 1
-----------TGGTTTTCACAATGATCGTCC-- t0816411 1
-----------TGGTTTTCACAATGATCGTCCA--- t0023829 45
-----------TGGTTTTCACAATGATCGTCCAG-- t0037108 27
-----------TGGTTTTCACAATGATCGTCCAGA--- t0093179 9
------------GGTTTTCACAATGATCGTCC-- t1874106 1
------------GGTTTTCACAATGATCGTCCA--- t0962557 1
------------GGTTTTCACAATGATCGTCCAG-- t0081904 11
------------GGTTTTCACAATGATCGTCCAGA--- t0238880 3
---------------TTTCACAATGATCGTCCA--- t0907488 1
---------------TTTCACAATGATCGTCCAG-- t2000047 1
---------------TTTCACAATGATCGTCCAGA--- t1637467 1
//
mireap
maleLPS-m0003 DS633978:113214:113295:+ 82(nt) -31.10(kcal/mol)
GCCTTCCGTTTTTTGGCACTAGCACATTTTTGTGTTTCGATGCTACGACAAAAATTGTGGTAGTGTCAAGCAATAGGAAGAG
maleLPS-m0003 26
..(((((((.(((((((((.((((((((((((.........)).))))))).)))).))))))))))).))).)))))..
AAAAATTGTGGTAGTGTCAAGCA*******
maleLPS-m0003-3p 26
--CAAAAATTGTGGTAGTGTCAA-------------

t1917118 1
--CAAAAATTGTGGTAGTGTCAAG------------
t1227458 1
---AAAAATTGTGGTAGTGTCAA-------------
t1630467 1
---AAAAATTGTGGTAGTGTCAAG------------
t0490562 2
---AAAAATTGTGGTAGTGTCAAGC-----------
t0170530 5
---AAAAATTGTGGTAGTGTCAAGCA----------
t0064070 15
--AAAATTGTGGTAGTGTCAAGCA----------
t0903424 1
//
mireap
maleLPS-m0004 DS685968:289:368:+ 80(nt) -33.00(kcal/mol)
GCTTAGGTGGGGCAGTGGAACTGTAGGGGTTACCACCGCAAACGCGGGGCGCTTACACAGATTCTCGCCACACAAGAGCA maleLPS-
m0004 6
((((..(((.(((.(.(((.((((..((((..((.((((....)))))).))))..)))).))).)))).)))..)))).
**********GGCAGTGGAACTGTAGGGGTTAC*** maleLPS-
m0004-5p 6
----------GGCAGTGGAACTGTAGGGGTT--- t1637306
1
----------GGCAGTGGAACTGTAGGGGTTA-- t0449339
2
----------GGCAGTGGAACTGTAGGGGTTAC--- t0268085
3
//
mireap
maleLPS-m0005 DS709336:35602:35671:+ 70(nt) -19.10(kcal/mol)
ATGGAGGACAGCACTCTTAAAAGCGCCGAGAATGCACGCGGCCCGTTGGTCTAGGGGTATGATTCTCGCT maleLPS-m0005 6
...(((((((..(((((((..(((((((.(......).))))..)))....))))))).)).)))))...
***************************************GGCCCGTTGGTCTAGGGGTAT********** maleLPS-m0005-3p 6
---------------------------------------GGCCCGTTGGTCTAGGGGTAT---------- t0133530 6
//
mireap
maleLPS-m0006 DS728967:139938:140028:- 91(nt) -28.30(kcal/mol)
CGCGAGAGCACTTGAATACCGCTAGCAACTTGTTTTATGATGTGAACTTCATCATCCATCGGCCCGTTGGTCTAGGGGTATGATTCTCGC
T maleLPS-m0006 6
.(((((((.......(((((.((((((((..(((....((((((.....)).))))....)))..))))..)))).)))))..))))))).
GGCCCGTTGGTCTAGGGGTAT*******
* maleLPS-m0006-3p 6
--GGCCCGTTGGTCTAGGGGTAT---------
- t0133530 6
//

mireap
maleLPS-m0007 DS767867:1053:1145:+ 93(nt) -30.00(kcal/mol)
GTGTCGACGAGGACACCGCCATGCTGCAGACCGTGACAACACACAAGGGCCTGTTCAAGGTGACGCGATTGCAGTTTGGTGTGGCAGTGG
CAG maleLPS-m0007 5
.((((.((...(.(((.((((.(((((((.((((.((.....(((......))).....)).))).).))))))).))))))).).))))
)).
CGCGATTGCAGTTTGGTGTG****
*** maleLPS-m0007-3p 5
---CGCGATTGCAGTTTGGTG---------
--- t2242017 1
---CGCGATTGCAGTTTGGTGTG-------
--- t0509649 2
---CGCGATTGCAGTTTGGTGTGG------
--- t1436138 1
---CGCGATTGCAGTTTGGTGTGGC-----
--- t1275246 1
//
mireap
maleLPS-m0008 DS788192:324939:325025:+ 87(nt) -46.00(kcal/mol)
TTGACGCCCTGTGCCGTCATCTGTCGTGTAGCGTAGGAACGCAGCAACGGCGAGCACGGCCGGACTGGAGAGGCGCGTGGGCGTCAC
maleLPS-m0008 10
.((((((((((((((.(((((((((((((.((((....)))).((....))..)))))).)))).)))...)))))).)))))))).
ACGGCCGGACTGGAGAGGCGCG*******
maleLPS-m0008-3p 10
---GCACGGCCGGACTGGAGAGGCG------------
t0912445 1
--CACGGCCGGACTGGAGAGGCG------------
t1883531 1
--CACGGCCGGACTGGAGAGGCGC-----------
t0805766 1
---ACGGCCGGACTGGAGAGGCGC-----------
t1161254 1
---ACGGCCGGACTGGAGAGGCGCG----------
t0157728 5
---GGCCGGACTGGAGAGGCGCG----------
t1306376 1
//
mireap
maleLPS-m0009 DS824332:107859:107942:+ 84(nt) -22.20(kcal/mol)
GTAACCAAGGTGTCCAGACGTCGGGTCGTCGTGACATGCTTGAAAGATCTCCTCTCGAAGGCCAGTCGGGATTACCAGCAGCCA
maleLPS-m0009 6
........(((((((.(((....((((.(((.((....((....))......)).))).)))).))).))).))))........
**********TGTCCAGACGTCGGGTCGTCGTG***
maleLPS-m0009-5p 6
---------GTGTCCAGACGTCGGGTCG--

t0702667 1
----------TGTCCAGACGTCGGGTCGTCGT--
t0405506 2
----------TGTCCAGACGTCGGGTCGTCGTG---
t0287005 3
//
mireap
maleLPS-m0010 DS845963:700:788:+ 89(nt) -20.30(kcal/mol)
TTGAGTAATGGCAGGTGAGGCTGATGTAACTTTGGTTAGACCTCGATGTTCATATGTCACTTGTAAACAGTCATGTACACGTTACTAAA
maleLPS-m0010 7
...(((((((((((((((.(...(((.(((...((.....)).....)))))).).))))))))..(((....)))...)))))))...
**********GCAGGTGAGGCTGATGTAACT**
maleLPS-m0010-5p 7
----------GCAGGTGAGGCTGATGTAACT--
t0145562 6
----------GCAGGTGAGGCTGATGTAACTT---
t0835497 1
//
mireap
maleLPS-m0011 DS878697:152111:152195:- 85(nt) -36.80(kcal/mol)
TTGCCAGTCGGGCGCAGTACCTCCAGGGACAGATCTGCATAGCCTGCTCGTTCCAGTATCCTGTCGGCTGCGCCACGACTCGCGC
maleLPS-m0011 8
..((.((((((((((((..((..(((((((.((...(((.....)))....))..)).)))))..))))))))).))))).))..
GTATCCTGTCGGCTGCGCCA*******
maleLPS-m0011-3p 8
---GTATCCTGTCGGCTGCGCCA----------
t0147613 6
--TATCCTGTCGGCTGCGCCA----------
t0711017 1
---ATCCTGTCGGCTGCGCCA----------
t0676520 1
//
mireap
maleLPS-m0012 DS904559:10:88:- 79(nt) -22.30(kcal/mol)
GTAATACTTTTCACGAATAAGGGCCAGCGTGTGACTGTATACAAGGTGTCCAGACGTCGGGTCGTCGTGGCAGGATTGA maleLPS-m0012 6
.((((.(((.((((((....((.((.((((.((((............)).)).)))).)).)).)))))).))))))).
TGTCCAGACGTCGGGTCGTCGTG******** maleLPS-m0012-3p 6
---GTGTCCAGACGTCGGGTCG--------------- t0702667 1
--TGTCCAGACGTCGGGTCGTCGT----------- t0405506 2
--TGTCCAGACGTCGGGTCGTCGTG---------- t0287005 3
//
mireap
maleLPS-m0013 DS908416:537143:537221:- 79(nt) -21.30(kcal/mol)

CCGACGATGACGATGACGACGACGACGATGCGTGTGCGCTGCTCGGCTACGACATCGCTACGGTGTGTGTATCGTGCGC maleLPS-m0013 17
.((((((((.((...(((.((..(.(((((((((.((.......)))))).))))))..)).))).)))))))).)).
**********CGATGACGACGACGACGATGCG*** maleLPS-m0013-5p 17
--------GACGATGACGACGACGACGAT-- t1033724 1
--------GACGATGACGACGACGACGATG--- t0876605 1
----------CGATGACGACGACGACGAT-- t1386834 1
----------CGATGACGACGACGACGATG--- t0383488 2
----------CGATGACGACGACGACGATGCG--- t0116661 7
-----------GATGACGACGACGACGATGCG--- t0195862 4
------------ATGACGACGACGACGATGC-- t1504949 1
//
mireap
maleLPS-m0014 DS911923:134628:134708:- 81(nt) -36.00(kcal/mol)
CACCAAAACCCTCTCTGTGCTGTGGAGGTAATATATAGCTGTCAACATATATACGTCCAAAGCACTGAGGGGGTTTTACTC maleLPS-m0014 7
....(((((((((((.(((((.((((.(((.(((((.........)))))))).)))).))))).)))))))))))....
TATACGTCCAAAGCACTGAGGG******** maleLPS-m0014-3p 7
---ATATACGTCCAAAGCACT--------------- t2228539 1
--TATACGTCCAAAGCACTG-------------- t0544244 2
--TATACGTCCAAAGCACTGAG------------ t0331722 2
--TATACGTCCAAAGCACTGAGGG---------- t0381439 2
//
