# Supplementary material for: Lipopolysaccharide-Induced Differential Expression of miRNAs in Male and Female Rhipicephalus haemaphysaloides Ticks
Source: PLoS One. 2015 Oct 2;10(10):e0139241. doi: 10.1371/journal.pone.0139241 (PMC4592253; doi:10.1371/journal.pone.0139241)
Supplement: S3 Text — (PDF) [file pone.0139241.s013.pdf]

mireap
PBS-m0001 DS633978:93549:93634:+ 86(nt) -41.60(kcal/mol)
CCTACCTTCCGTCAATGGCACTGGAAGAATTCACGGGGTCTTGATAGAAATACCGTGACTTCTCCGGTGCTGTGGATGGCGGCTAG PBS-m0001 40
.(((.((.(((((.(((((((((((.(((.((((((..(((....)))....)))))).)))))))))))))).))))).)).)))
GTGACTTCTCCGGTGCTGTGGA******** PBS-m0001-3p 40
---CGTGACTTCTCCGGTGCT--------------- t0203515 4
---CGTGACTTCTCCGGTGCTGTG------------ t0951303 1
---CGTGACTTCTCCGGTGCTGTGGA---------- t1393361 1
--GTGACTTCTCCGGTGCTG-------------- t0092866 8
--GTGACTTCTCCGGTGCTGT------------- t0234176 3
--GTGACTTCTCCGGTGCTGTG------------ t0240079 3
--GTGACTTCTCCGGTGCTGTGG----------- t0121174 6
--GTGACTTCTCCGGTGCTGTGGA---------- t0060550 13
---TGACTTCTCCGGTGCTGTGGA---------- t0997371 1
//
mireap
PBS-m0002 DS633978:113214:113295:+ 82(nt) -31.10(kcal/mol)
GCCTTCCGTTTTTTGGCACTAGCACATTTTTGTGTTTCGATGCTACGACAAAAATTGTGGTAGTGTCAAGCAATAGGAAGAG PBS-m0002 35
..((((((((.((((((((((.(((((((((((((.........)).))))))).)))).))))))))))).))).)))))..
AAAAATTGTGGTAGTGTCAAGCA******* PBS-m0002-3p 35
--CAAAAATTGTGGTAGTGTCAAG------------ t0364915 2
--CAAAAATTGTGGTAGTGTCAAGC----------- t0142974 5
--CAAAAATTGTGGTAGTGTCAAGCA---------- t0409914 2
---AAAAATTGTGGTAGTGTCAA------------- t0410497 2
---AAAAATTGTGGTAGTGTCAAG------------ t0438727 2
---AAAAATTGTGGTAGTGTCAAGC----------- t0414649 2
---AAAAATTGTGGTAGTGTCAAGCA---------- t0047990 17
--AAAATTGTGGTAGTGTCAAGCA---------- t0222646 3
//
mireap
PBS-m0003 DS670462:6372:6469:- 98(nt) -22.60(kcal/mol)
GCCATGGCGACGTATCACGTCCGGGTCACCAATTGTAGAGACACAAGAATTGGCCGACCGACTGTTACGATAAGAAAAGCATTTATAGAATCGCCCGT
PBS-m0003 10
.....((((((((.((.(((..((((.((((((.(.(.....).).))))))..)))))))).))))....(.....)..........)))))...
**********CGTATCACGTCCGGGTCACCA***
PBS-m0003-5p 10
----------CGTATCACGTCCGGGTCACCA---
t0157298 5
-----------GTATCACGTCCGGGTCACCA---
t0467083 2
------------TATCACGTCCGGGTCACCA---
t0234722 3
//
mireap
PBS-m0004 DS682300:151461:151545:- 85(nt) -37.30(kcal/mol)
TGCCGACCTATGTTGATCGGGTGTCCGCGCTATGCTGGGCGTCGATATGGTGACCCCAGGCGACCCCGGGGCGACCAGGTCGCCT PBS-m0004 10
.(.((((((..((((.(((((.(((.((......(((((.((((......)))))))))))))))))))..)))).)))))).).
**********TGTTGATCGGGTGTCCGCGCTA*** PBS-m0004-5p

9
--------TATGTTGATCGGGTGTCC--- t1146177 1
--------TATGTTGATCGGGTGTCCGCGCT-- t2079422 1
---------ATGTTGATCGGGTGTCCGC--- t0836426 1
----------TGTTGATCGGGTGTCCGCGC--- t2100160 1
----------TGTTGATCGGGTGTCCGCGCTA--- t0257308 3
----------TGTTGATCGGGTGTCCGCGCTAT-- t1152430 1
------------TTGATCGGGTGTCCGCGCTA--- t0607937 1
---------------------------------GCTGGGCGTCGATATGGTG--------------------------------- t1050284 1
//
mireap
PBS-m0005 DS696092:157396:157474:+ 79(nt) -45.64(kcal/mol)
TGGCGGACTCTAAGTTAATCTCCAAGCCCAATGGTAATTCTCGTCAATTGGGCTTGAAGATTAGCTTAGACCCCGCCAA PBS-m0005 12
((((((..(((((((((((((.((((((((((..............)))))))))).)))))))))))))..)))))).
**********TAAGTTAATCTCCAAGCCCAAT*** PBS-m0005-5p 12
---------CTAAGTTAATCTCCAAGCCCAAT--- t0863468 1
----------TAAGTTAATCTCCAAGCCCA--- t0334496 2
----------TAAGTTAATCTCCAAGCCCAA-- t0363149 2
----------TAAGTTAATCTCCAAGCCCAAT--- t0121719 6
----------TAAGTTAATCTCCAAGCCCAATG-- t1700189 1
//
mireap
PBS-m0006 DS709336:35602:35671:+ 70(nt) -19.10(kcal/mol)
ATGGAGGACAGCACTCTTAAAAGCGCCGAGAATGCACGCGGCCCGTTGGTCTAGGGGTATGATTCTCGCT PBS-m0006 6
...(((((((..((((((((..(((((((.(......).))))..)))....))))))).)).))))))...
***************************************GGCCCGTTGGTCTAGGGGTAT********** PBS-m0006-3p 6
---------------------------------------GGCCCGTTGGTCTAGGGGTAT---------- t0117485 6
//
mireap
PBS-m0007 DS728967:139938:140028:- 91(nt) -28.30(kcal/mol)
CGCGAGAGCACTTGAATACCGCTAGCAACTTGTTTTATGATGTGAACTTCATCATCCATCGGCCCGTTGGTCTAGGGGTATGATTCTCGCT PBS-m0007 6
.(((((((.......(((((.((((((((..(((....((((((.....)).))))....)))..))))..)))).)))))..))))))).
GGCCCGTTGGTCTAGGGGTAT******** PBS-m0007-3p 6
--GGCCCGTTGGTCTAGGGGTAT----------
t0117485 6
//
mireap
PBS-m0008 DS762741:4587:4681:+ 95(nt) -18.60(kcal/mol)
ACGCATCATGATCCTCATGCTGCTGTCTCTTATGATGATTTTGAGTAGGACTATGATGATAATGACGATGACGACGACGACGATGATGATACGCG
PBS-m0008 11
.((((((((.(((.((....((.((((((((((.((.(((...((.....))..))).)).)))).)).)))).))..)).))).)))))..)))
GACGATGACGACGACGACGATG*******
PBS-m0008-3p 11
---ATGACGATGACGACGACGACGATGA---------
t1847869 1
---GACGATGACGACGACGACGAT-----------
t0228208 3

---GACGATGACGACGACGACGATG----------
t0118268 6
---GACGATGACGACGACGACGATGA---------
t1594873 1
//
mireap
PBS-m0009 DS805199:562:650:- 89(nt) -20.90(kcal/mol)
GCCATAGCGACGTATCACGTCCGGGTCACCAATTGTAGAGACACAAGAATTGGCCGACCGACTGTTACGATAAGAAAAGCATTTATGGA PBS-m0009
10
.((((((...((((.((.(((..((((.((((((.(.(.....).).))))))..))))))))).))))....(.....)..)))))).
**********CGTATCACGTCCGGGTCACCA** PBS-
m0009-5p 10
----------CGTATCACGTCCGGGTCACCA-- t0157298
5
-----------GTATCACGTCCGGGTCACCA-- t0467083
2
------------TATCACGTCCGGGTCACCA-- t0234722
3
//
mireap
PBS-m0010 DS834826:12626:12709:+ 84(nt) -37.60(kcal/mol)
CTCCCTACTGCAGGTGAGAGGACATAGCCAGTGCTGTTATTTTCGATTGGCAGTGGTCATGTCTTCGCACTGGACGGTGGTGAA PBS-m0010 121
.((.(((((((.((((.((((((((..(((.(((((...........))))).)))..)))))))).)))).).)))))).)).
AGTGGTCATGTCTTCGCACTGGA******* PBS-m0010-3p
121
---AGTGGTCATGTCTTCGCA--------------- t0460648 2
---AGTGGTCATGTCTTCGCAC-------------- t0128599 6
---AGTGGTCATGTCTTCGCACT------------- t0147700 5
---AGTGGTCATGTCTTCGCACTG------------ t0032349 25
---AGTGGTCATGTCTTCGCACTGG----------- t0027733 30
---AGTGGTCATGTCTTCGCACTGGA---------- t0016076 53
//
mireap
PBS-m0011 DS845963:700:788:+ 89(nt) -20.30(kcal/mol)
TTGAGTAATGGCAGGTGAGGCTGATGTAACTTTGGTTAGACCTCGATGTTCATATGTCACTTGTAAACAGTCATGTACACGTTACTAAA PBS-m0011
11
...(((((((((((((((.(...(((.(((...((.....)).....)))))).).))))))))..(((....)))...)))))))...
**********GCAGGTGAGGCTGATGTAACTT*** PBS-
m0011-5p 11
----------GCAGGTGAGGCTGATGTAACT-- t0193664
4
----------GCAGGTGAGGCTGATGTAACTT--- t0102462
7
//
mireap
PBS-m0012 DS855459:689:769:- 81(nt) -24.40(kcal/mol)
CTCGGTCCGTAATCAGGACTTCGGGGGCGCCGTGTCGGAGGACGATAGACTCAACGAAGAACTTGGCTACCACGACCCGAA PBS-m0012 5
.((((((.(((..((((.(((((..((.(.(.(((((.....)))))).)))..)))))..))))..)))...)).))))).
CTCAACGAAGAACTTGGCTACC******* PBS-m0012-3p 5

---CTCAACGAAGAACTTGGCTA------------ t0876177 1
---CTCAACGAAGAACTTGGCTAC----------- t0358154 2
---CTCAACGAAGAACTTGGCTACC---------- t0298236 2
//
mireap
PBS-m0013 DS862621:188:285:+ 98(nt) -21.10(kcal/mol)
GCCATAGCGACGTATCACGTCCGGGTCACCAATTGTAGAGACACAAGAATTGGCCGACCGACTGTTACGATAAGAAAAGCATTTGTAGAATCGCCCGT
PBS-m0013 10
......((((((((.((.(((..((((.((((((.(.(.....).).))))))..))))))))).)))).........((....))....))))....
**********CGTATCACGTCCGGGTCACCA**
PBS-m0013-5p 10
----------CGTATCACGTCCGGGTCACCA--
t0157298 5
-----------GTATCACGTCCGGGTCACCA--
t0467083 2
------------TATCACGTCCGGGTCACCA--
t0234722 3
//
mireap
PBS-m0014 DS911299:1700299:1700382:- 84(nt) -42.70(kcal/mol)
GGAGGGCCTTCCTCACTCAGTTTGGCTGTGGTGTAACGGGCGCTCGACCCATCACAACCTCCTTGAGTGAGTGAGGCCTCGCCG PBS-m0014 138
((.((((((..(((((((((...((.(((((((...(((....)))...))))))).))...)))))))))..))))))..)).
**********CCTCACTCAGTTTGGCTGTG** PBS-m0014-5p
136
----------CCTCACTCAGTTTGGCTG-- t0077230 10
----------CCTCACTCAGTTTGGCTGT--- t0030082 27
----------CCTCACTCAGTTTGGCTGTG-- t0025117 33
----------CCTCACTCAGTTTGGCTGTGG--- t0029234 28
----------CCTCACTCAGTTTGGCTGTGGT-- t0026135 32
----------CCTCACTCAGTTTGGCTGTGGTG--- t0205031 4
------------TCACTCAGTTTGGCTGTGGTGT-- t0394337 2
----------------TCAGTTTGGCTGTGGTGTAAC--- t2019881 1
--------------------TTTGGCTGTGGTGTAACGG--- t1204236 1
//
mireap
PBS-m0015 DS911923:134629:134707:- 79(nt) -36.00(kcal/mol)
ACCAAAACCCTCTCTGTGCTGTGGAGGTAATATATAGCTGTCAACATATATACGTCCAAAGCACTGAGGGGGTTTTACT PBS-m0015 49
...(((((((((((.(((((.((((.(((.(((((..........)))))))).)))).))))).)))))))))))...
TATACGTCCAAAGCACTGAGG******** PBS-m0015-3p 49
---ATATACGTCCAAAGCACT-------------- t1903199 1
---ATATACGTCCAAAGCACTGAGG---------- t1995622 1
--TATACGTCCAAAGCACTGA------------ t1835105 1
--TATACGTCCAAAGCACTGAG----------- t0062637 12
--TATACGTCCAAAGCACTGAGG---------- t0036905 22
--TATACGTCCAAAGCACTGAGGG--------- t0082855 9
--TATACGTCCAAAGCACTGAGGGG-------- t0247939 3
//
mireap
PBS-m0016 DS918772:579:661:+ 83(nt) -24.90(kcal/mol)

```
GCCGTAGCCGCGTATCACGTCCGGGTCACCAATTGTAGAGACACAAGAATTGGTCGACCGACTGGTATGATAAAAACTGCATT PBS-m0016 10
...((((...((((((.(((..((((((((((((.(.(.....).).))))))).)))))))))))))).......))))...
**********CGTATCACGTCCGGGTCACCA**************************************************** PBS-m0016-5p 10
----------CGTATCACGTCCGGGTCACCA---------------------------------------------------- t0157298 5
-----------GTATCACGTCCGGGTCACCA---------------------------------------------------- t0467083 2
------------TATCACGTCCGGGTCACCA---------------------------------------------------- t0234722 3
//
mireap
PBS-m0017 DS966815:32756:32835:- 80(nt) -36.26(kcal/mol)
CCGAGTGCTGACTCGAGCTGCCCGTGCAAAACTGGAACCACTGCGTTGTTTTGTTCGTTCGGCTCGAGTTAGAAACCGGT PBS-m0017 61
(((.((.(((((((((((((..((.(((((((..............))))))).))..)))))))))))))..))))).
**********ACTCGAGCTGCCCGTGCAAAAC************************************************ PBS-m0017-5p 61
---------GACTCGAGCTGCCCGTGCAAA-------------------------------------------------- t0778925 1
----------ACTCGAGCTGCCCGTGCAA--------------------------------------------------- t0145312 5
----------ACTCGAGCTGCCCGTGCAAA-------------------------------------------------- t0129392 6
----------ACTCGAGCTGCCCGTGCAAAA------------------------------------------------- t0064320 12
----------ACTCGAGCTGCCCGTGCAAAAC------------------------------------------------ t0029865 27
----------ACTCGAGCTGCCCGTGCAAAACT----------------------------------------------- t0078538 10
//
mireap
PBS-m0018 DS980795:53:140:+ 88(nt) -32.70(kcal/mol)
TTGCACTTTTCGAATCCCATCCTCGTCGCCATTTTGTATTTTTGAGGAGGCCGATGTTGGTAGTGAGGTGGTTGTCCGAAAGGTGCTG PBS-m0018
20
..((((((((((.((.(((.(((((..((((..(((..(((.....)))..)))...))))..))))))))..)).))))))))))..
**********CGAATCCCATCCTCGTCGCCA********************************************************* PBS-m0018-
5p 20
--------TTCGAATCCCATCCTCGTCGCCA--------------------------------------------------------- t0360062 2
---------TCGAATCCCATCCTCGTCGCCA--------------------------------------------------------- t0160412 5
----------CGAATCCCATCCTCGTCGCCA--------------------------------------------------------- t0060377
13
//
```
