# Supplementary material for: Lipopolysaccharide-Induced Differential Expression of miRNAs in Male and Female Rhipicephalus haemaphysaloides Ticks
Source: PLoS One. 2015 Oct 2;10(10):e0139241. doi: 10.1371/journal.pone.0139241 (PMC4592253; doi:10.1371/journal.pone.0139241)
Supplement: S4 Text — (PDF) [file pone.0139241.s014.pdf]

mireap
LPS-m0001 DS633978:93549:93634:+ 86(nt) -41.60(kcal/mol)
CCTACCTTCCGTCAATGGCACTGGAAGAATTCACGGGGTCTTGATAGAAATACCGTGACTTCTCCGGTGCTGTGGATGGCGGCTAG
LPS-m0001 35
.(((.((.(((((.(((((((((((.(((.((((((..(((....)))....)))))).)))))))))))))).))))).)).)))
GTGACTTCTCCGGTGCTGTGGA********
LPS-m0001-3p 35
---CGTGACTTCTCCGGTGCT---------------
t0505617 2
---CGTGACTTCTCCGGTGCTGTG------------
t0991896 1
---CGTGACTTCTCCGGTGCTGTGGA----------
t0428844 2
--GTGACTTCTCCGGTGCTG--------------
t0372286 2
--GTGACTTCTCCGGTGCTGT-------------
t0151634 5
--GTGACTTCTCCGGTGCTGTG------------
t0098995 8
--GTGACTTCTCCGGTGCTGTGGA----------
t0059777 14
---TGACTTCTCCGGTGCTGTGGA----------
t1039488 1
//
mireap
LPS-m0002 DS633978:113214:113295:+ 82(nt) -31.10(kcal/mol)
GCCTTCCGTTTTTTGGCACTAGCACATTTTTGTGTTTCGATGCTACGACAAAAATTGTGGTAGTGTCAAGCAATAGGAAGAG LPS-m0002 36
..((((((((.((((((((((.(((((((((((((.........)).))))))).)))).)))))))))).))).)))))..
AAAAATTGTGGTAGTGTCAAGCA******* LPS-m0002-3p 36
--CAAAAATTGTGGTAGTGTCAAG------------
t1104339 1
--CAAAAATTGTGGTAGTGTCAAGC-----------
t1012867 1
--CAAAAATTGTGGTAGTGTCAAGCA----------
t0429159 2
---AAAAATTGTGGTAGTGTCAAGC-----------
t0133312 6
---AAAAATTGTGGTAGTGTCAAGCA----------
t0039751 22
---AAAAATTGTGGTAGTGTCAAGCAA---------
t1729819 1
--AAAATTGTGGTAGTGTCAAGCA----------
t0234206 3

//
mireap
LPS-m0003 DS668020:5090:5175:- 86(nt) -42.40(kcal/mol)
CATGGATAGCACGGCCGGACTGGAGAGGCGCGGGCGTCACAAGCCTGCGCCTCTCTACATACGGGGGTGCTTAAATTCACATGC
LPS-m0003 11
((((((.(((((..(((...(((((((((((..((((.......)))))))))))))))....)))..))))).....)).)))).
**********ACGGCCGGACTGGAGAGGCGCG**
LPS-m0003-5p 11
---------CACGGCCGGACTGGAGAGGCG--
t0459257 2
----------ACGGCCGGACTGGAGAGGCG--
t1174880 1
----------ACGGCCGGACTGGAGAGGCGCG--
t0147183 5
----------ACGGCCGGACTGGAGAGGCGCGC---
t0254069 3
//
mireap
LPS-m0004 DS696092:157396:157474:+ 79(nt) -45.64(kcal/mol)
TGGCGGACTCTAAGTTAATCTCCAAGCCCAATGGTAATTCTCGTCAATTGGGCTTGAAGATTAGCTTAGACCCCGCCAA LPS-m0004
10
((((((..(((((((((((((.((((((((((...............)))))))))).)))))))))))))..)))))).
**********TAAGTTAATCTCCAAGCCCAAT*** LPS-m0004-
5p 10
----------TAAGTTAATCTCCAAGCC--- t0589191 1
----------TAAGTTAATCTCCAAGCCCAA-- t0248041 3
----------TAAGTTAATCTCCAAGCCCAAT--- t0128398 6
//
mireap
LPS-m0005 DS709336:35602:35671:+ 70(nt) -19.10(kcal/mol)
ATGGAGGACAGCACTCTTAAAAGCGCCGAGAATGCACGCGGCCCGTTGGTCTAGGGGTATGATTCTCGCT LPS-m0005 6
...(((((((..(((((((..(((((((.(......).))))..)))....))))))).)).)))))...
***************************************GGCCCGTTGGTCTAGGGGTAT********** LPS-m0005-3p 6
---------------------------------------GGCCCGTTGGTCTAGGGGTAT---------- t0123899 6
//
mireap
LPS-m0006 DS728967:139938:140028:- 91(nt) -28.30(kcal/mol)
CGCGAGAGCACTTGAATACCGCTAGCAACTTGTTTTATGATGTGAACTTCATCATCCATCGGCCCGTTGGTCTAGGGGTATGATTCTCGC
T LPS-m0006 6
.(((((((.......(((((.((((((((..(((....((((((.....)).))))....)))..))))..)))).)))))..))))))).
GGCCCGTTGGTCTAGGGGTAT*******
* LPS-m0006-3p 6
--GGCCCGTTGGTCTAGGGGTAT---------
- t0123899 6
//

mireap
LPS-m0007 DS768911:796132:796218:+ 87(nt) -20.90(kcal/mol)
TTCCAGGTTATACCGTTAGGTTGGATTAGCTTAGGCAAGGTTAGGCTAGGTTAGGTTAGGTTAGGTTAGGTTGGGTTTTATTTGTAA
LPS-m0007 6
.(((((.(((.(((..(((.((((.(((((((((.(.......).))))))))).)))).))))))))).)))))............
TAGGTTAGGTTAGGTTGGGTT********
LPS-m0007-3p 6
---TTAGGTTAGGTTAGGTTGGGTT----------
t1665599 1
--TAGGTTAGGTTAGGTTGG-------------
t1343107 1
--TAGGTTAGGTTAGGTTGGG------------
t1526206 1
--TAGGTTAGGTTAGGTTGGGTT----------
t0252364 3
//
mireap
LPS-m0008 DS788192:324939:325025:+ 87(nt) -46.00(kcal/mol)
TTGACGCCCTGTGCCGTCATCTGTCGTGTAGCGTAGGAACGCAGCAACGGCGAGCACGGCCGGACTGGAGAGGCGCGTGGGCGTCAC
LPS-m0008 8
.((((((((((((((.(((((((((((((.((((....)))).((....))..)))))).)))).)))...)))))).)))))))).
ACGGCCGGACTGGAGAGGCGCG*******
LPS-m0008-3p 8
--CACGGCCGGACTGGAGAGGCG------------
t0459257 2
---ACGGCCGGACTGGAGAGGCG------------
t1174880 1
---ACGGCCGGACTGGAGAGGCGCG----------
t0147183 5
//
mireap
LPS-m0009 DS790259:165907:165994:- 88(nt) -22.70(kcal/mol)
GCGGTGCCCTCTTCAGCCGTCCGGTCGGCGTCTGCTCATGGCTGCCGCTTGCCCTCCATTCAGTGTAGAGCCTTCGGAATTCAACCTT
LPS-m0009 13
(((((..........)))))((((..(((.(((((.(((((..((.....))...))))...).)))))))).))))...........
TCCATTCAGTGTAGAGCCTTCGGA********
LPS-m0009-3p 13
--TCCATTCAGTGTAGAGCCTTCGGA----------
t0096875 8
--TCCATTCAGTGTAGAGCCTTCGGAA---------
t0193370 4
---CCATTCAGTGTAGAGCCTTCGG-----------
t0566323 1
//
mireap

LPS-m0010 DS803854:581:659:- 79(nt) -35.50(kcal/mol)
TTCCACGTTTCCTTATCATTCGACTGTCCAGACTGCTGTTTTGGAGACCTGGACGGAGAACTGATAAGGGCTTGTGGTT LPS-m0010 1153
..(((((..((((((((((((..((((((((.((.(......).))..)))))))).))).)))))))))..)))))..
**********CCTTATCATTCGACTGTCCAGA*** LPS-m0010-5p 1153
---------TCCTTATCATTCGACTGTCCAG-- t0414397 2
---------TCCTTATCATTCGACTGTCCAGA--- t0111759 7
----------CCTTATCATTCGACTGTCCA--- t0297336 3
----------CCTTATCATTCGACTGTCCAG-- t0005204 182
----------CCTTATCATTCGACTGTCCAGA--- t0000952 912
----------CCTTATCATTCGACTGTCCAGAC-- t0120306 7
----------CCTTATCATTCGACTGTCCAGACT--- t0293568 3
----------CCTTATCATTCGACTGTCCAGACTGC--- t0740131 1
-----------CTTATCATTCGACTGTCCAG-- t0141855 6
-----------CTTATCATTCGACTGTCCAGA--- t0047151 18
-----------CTTATCATTCGACTGTCCAGAC-- t1941979 1
------------TTATCATTCGACTGTCCAGA--- t0217635 4
------------TTATCATTCGACTGTCCAGAC-- t0515757 2
------------TTATCATTCGACTGTCCAGACT--- t0213128 4
-------------TATCATTCGACTGTCCAG-- t1878685 1
//
mireap
LPS-m0011 DS834826:12626:12709:+ 84(nt) -37.60(kcal/mol)
CTCCCTACTGCAGGTGAGAGGACATAGCCAGTGCTGTTATTTTCGATTGGCAGTGGTCATGTCTTCGCACTGGACGGTGGTGAA LPS-m0011 119
.((.(((((((.((((.((((((((..(((.(((((...........))))).)))..)))))))).)))).).)))))).)).
AGTGGTCATGTCTTCGCACTGGA******* LPS-m0011-3p 119
---AGTGGTCATGTCTTCGCA--------------- t0287246 3
---AGTGGTCATGTCTTCGCAC-------------- t0204583 4
---AGTGGTCATGTCTTCGCACT------------- t0130823 6
---AGTGGTCATGTCTTCGCACTG------------ t0044477 19
---AGTGGTCATGTCTTCGCACTGG----------- t0024146 37
---AGTGGTCATGTCTTCGCACTGGA---------- t0018069 50
//

mireap
LPS-m0012 DS845963:700:788:+ 89(nt) -20.30(kcal/mol)
TTGAGTAATGGCAGGTGAGGCTGATGTAACTTTGGTTAGACCTCGATGTTCATATGTCACTTGTAAACAGTCATGTACACGTTACTAAA
LPS-m0012 11
...(((((((((((((((.(...(((.(((...((.....)).....)))))).).))))))))..(((....)))...)))))))...
**********GCAGGTGAGGCTGATGTAACTT***
LPS-m0012-5p 11
----------GCAGGTGAGGCTGATGTAACT--
t0277483 3
----------GCAGGTGAGGCTGATGTAACTT---
t0108362 7
----------GCAGGTGAGGCTGATGTAACTTT--
t1010290 1
//
mireap
LPS-m0013 DS886901:16593:16670:- 78(nt) -35.70(kcal/mol)
CCGCACTCCCCTGGGAAGGTCTGAGACAGGAGCCCGCACAGGCTCAGGCCCTAGCAGGGTCCCGACCGAGAGGTCGGT LPS-m0013
162
........(((((..(.((((((((.(..(.(....).)..))))))))).)..)))))..((((((....)))))).
**********CTGGGAAGGTCTGAGACAGGAG** LPS-m0013-
5p 161
-----CTCCCCTGGGAAGGTCTGAGAC--- t0856916 1
------TCCCCTGGGAAGGTCTGAGACA-- t1761326 1
------TCCCCTGGGAAGGTCTGAGACAG--- t1677080 1
---------CCTGGGAAGGTCTGAGACAGGA--- t0526134 2
---------CCTGGGAAGGTCTGAGACAGGAG-- t0780292 1
----------CTGGGAAGGTCTGAGACAG--- t0519274 2
----------CTGGGAAGGTCTGAGACAGGA--- t0095820 8
----------CTGGGAAGGTCTGAGACAGGAG-- t0009609 97
-----------TGGGAAGGTCTGAGACAGGA--- t0251610 3
-----------TGGGAAGGTCTGAGACAGGAG-- t0032461 27
-----------TGGGAAGGTCTGAGACAGGAGC--- t0052714 16
--------------GAAGGTCTGAGACAGGAGC--- t0892656 1
--------------GAAGGTCTGAGACAGGAGCC-- t0501122 2
//
mireap
LPS-m0014 DS908416:537143:537221:- 79(nt) -21.30(kcal/mol)
CCGACGATGACGATGACGACGACGACGATGCGTGTGCGCTGCTCGGCTACGACATCGCTACGGTGTGTGTATCGTGCGC LPS-m0014
25
.(((((((.((...(((.((..(.(((((((((.((.......)))))).))))))..)).))).))))))).)).
**********CGATGACGACGACGACGATGCG*** LPS-m0014-
5p 25
--------GACGATGACGACGACGACGAT-- t0936066 1
--------GACGATGACGACGACGACGATG--- t0341475 2
----------CGATGACGACGACGACGAT-- t1243052 1

----------CGATGACGACGACGACGATGC-- t1648466 1
----------CGATGACGACGACGACGATGCG--- t0071952
11
-----------GATGACGACGACGACGATGCG--- t0110049 7
------------ATGACGACGACGACGATGC-- t1346225 1
--------------GACGACGACGACGATGCG--- t1239190 1
//
mireap
LPS-m0015 DS909479:5723:5809:+ 87(nt) -26.80(kcal/mol)
AGATTCGCTAAGCCTAGCTGAAGCCTAAACAATATTTCACGTGAAATTGTTTTTGTTAGGTTAGGTTAGGTTGGGTTAAGCACGCGG
LPS-m0015 6
......(((.(((((((((..(((((((((((.(((((....)))))....)))))....))))))).))))))))).)))......
TAGGTTAGGTTAGGTTGGGTT********
LPS-m0015-3p 6
---TTAGGTTAGGTTAGGTTGGGTT----------
t1665599 1
--TAGGTTAGGTTAGGTTGG-------------
t1343107 1
--TAGGTTAGGTTAGGTTGGG------------
t1526206 1
--TAGGTTAGGTTAGGTTGGGTT----------
t0252364 3
//
mireap
LPS-m0016 DS911299:1700299:1700382:- 84(nt) -42.70(kcal/mol)
GGAGGGCCTTCCTCACTCAGTTTGGCTGTGGTGTAACGGGCGCTCGACCCATCACAACCTCCTTGAGTGAGTGAGGCCTCGCCG LPS-
m0016 163
((.((((((..(((((((((...((.(((((((...(((....)))...))))))).))...)))))))))..))))))..)).
**********CCTCACTCAGTTTGGCTGTG** LPS-
m0016-5p 162
----------CCTCACTCAGTTTGGCTG--
t0049520 17
----------CCTCACTCAGTTTGGCTGT---
t0037148 23
----------CCTCACTCAGTTTGGCTGTG--
t0019925 45
----------CCTCACTCAGTTTGGCTGTGG---
t0028199 31
----------CCTCACTCAGTTTGGCTGTGGT--
t0020945 43
----------CCTCACTCAGTTTGGCTGTGGTG---
t0299269 3
----------------TCAGTTTGGCTGTGGTGTAACGGG--
t0625127 1
//

mireap
LPS-m0017 DS911923:134629:134707:- 79(nt) -36.00(kcal/mol)
ACCAAAACCCTCTCTGTGCTGTGGAGGTAATATATAGCTGTCAACATATATACGTCCAAAGCACTGAGGGGGTTTTACT LPS-m0017
61
...((((((((((.(((((.((((.(((.(((((.........)))))))).)))).))))).)))))))))))...
TATACGTCCAAAGCACTGAGG******** LPS-m0017-
3p 61
---ATATACGTCCAAAGCACT-------------- t1975392 1
---ATATACGTCCAAAGCACTGA------------ t1780169 1
---ATATACGTCCAAAGCACTGAGG---------- t2070640 1
--TATACGTCCAAAGCACTG------------- t2042409 1
--TATACGTCCAAAGCACTGA------------ t0290202 3
--TATACGTCCAAAGCACTGAG----------- t0066206
12
--TATACGTCCAAAGCACTGAGG---------- t0027527
32
--TATACGTCCAAAGCACTGAGGG--------- t0097206 8
--TATACGTCCAAAGCACTGAGGGG-------- t1333389 1
---ATACGTCCAAAGCACTGAG----------- t1996704 1
//
mireap
LPS-m0018 DS966815:32756:32835:- 80(nt) -36.26(kcal/mol)
CCGAGTGCTGACTCGAGCTGCCCGTGCAAAACTGGAACCACTGCGTTGTTTGTTCGTTCGGCTCGAGTTAGAAACCGGT LPS-m0018
55
(((.((.((((((((((((..((.(((((((...............))))))).))..))))))))))))..))))).
**********ACTCGAGCTGCCCGTGCAAAACT*** LPS-
m0018-5p 55
----------ACTCGAGCTGCCCGTGCAA--- t0063197
13
----------ACTCGAGCTGCCCGTGCAAA-- t0117335
7
----------ACTCGAGCTGCCCGTGCAAAA--- t0063119
13
----------ACTCGAGCTGCCCGTGCAAAAC-- t0086321
9
----------ACTCGAGCTGCCCGTGCAAAACT--- t0064610
13
//
mireap
LPS-m0019 DS971679:69442:69539:+ 98(nt) -21.30(kcal/mol)
AAAGCCGTTATGCTTCCCATGAGTTAGGCTATTTAGTTAGGTTAGGTTAGGTTAGGTTAGGTTAGGTTAGGTTAGGTTAGGTTGGGTTAG
TTAGGCTT LPS-m0019 6
.(((((.....(((.(((((((.((((.(((...(.((((.((((.(((...))).)))).)))).)))).)))).)))...)))))..))
)..)))))
***TAGGTTAGGTTAGGTTGGGTT**

******** LPS-m0019-3p 6
--TTAGGTTAGGTTAGGTTGGGTT--
-------- t1665599 1
---TAGGTTAGGTTAGGTTGG-----
-------- t1343107 1
---TAGGTTAGGTTAGGTTGGG----
-------- t1526206 1
---TAGGTTAGGTTAGGTTGGGTT--
-------- t0252364 3
//
mireap
LPS-m0020 DS980795:53:140:+ 88(nt) -32.70(kcal/mol)
TTGCACTTTTCGAATCCCATCCTCGTCGCCATTTTGTATTTTTGAGGAGGCCGATGTTGGTAGTGAGGTGGTTGTCCGAAAGGTGCTG
LPS-m0020 8
..((((((((((.((.(((.(((((..((((..(((..(((.....)))..)))...))))..))))))))..)).))))))))))..
**********CGAATCCCATCCTCGTCGCCA***
LPS-m0020-5p 8
--------TTCGAATCCCATCCTCGTCGCCA---
t0377500 2
---------TCGAATCCCATCCTCGTCGCCA---
t2043418 1
----------CGAATCCCATCCTCGTCGCCA---
t0157958 5
//
